# Causal relationships between inflammatory cytokines and myopia: an analysis of genetic and observational studies

**DOI:** 10.1097/MS9.0000000000002325

**Published:** 2024-07-02

**Authors:** Rongbin Liang, Tao Li, Hui Gao, Wenqing Shi, Meilin Li, Ting Wan, Xiaodong Zhou

**Affiliations:** a Department of Ophthalmology, Jinshan Hospital of Fudan University, Shanghai, China; b Department of Anatomy & Embryology, Maastricht University, Maastricht, The Netherlands

**Keywords:** genetics, inflammation, Mendelian randomization, myopia, vitreous

## Abstract

**Objective:**

This study aims to explore the causal relationship between inflammatory markers and myopia through the use of bidirectional Mendelian randomization (MR) and myopia animal models.

**Methods:**

The authors utilized data from a comprehensive and publicly accessible genome-wide association study (GWAS) for our analysis, which includes 460 536 European ancestry control subjects and 37 362 myopia patients. Utilizing a two-sample Mendelian randomization analysis framework, 27 inflammatory markers were investigated as exposure variables with myopia serving as the outcome variable. Nine MR analysis techniques were employed, with inverse-variance weighting (IVW) as the principal MR analysis method. Heterogeneity was assessed using Cochrane’s Q test. The identification of single-nucleotide polymorphisms (SNPs) and outliers linked to myopia was achieved via MR-PRESSO. The expression of interleukin-2 (IL-2) in the vitreous of guinea pigs subjected to experimentally induced form-deprivation myopia (FDM) was examined.

**Results:**

Elevated concentrations of IL-2 and IL-2ra were found to be associated [IVW estimate odds ratio (OR): 1.003, 95% CI: 1.001–1.005, *P*=0.001] and strongly associated (IVW estimate OR: 1.002, 95% CI: 1.000–1.003, *P*=0.049) with an increased risk of myopia, respectively. Conversely, lower levels of C-reactive protein (CRP) (IVW estimate OR: 0.996, 95% CI: 0.994–0.999, *P*=0.002) and tumour necrosis factor alpha (IVW estimate OR: 0.995, 95% CI: 0.994–0.996, *P*<0.001) were robustly linked to a heightened risk of myopia. IL-2 expression was notably upregulated in the vitreous of guinea pigs with experimentally induced FDM.

**Conclusions:**

Elevated levels of inflammatory factors, especially IL-2 and IL-2ra, have a potential causal relationship with myopia susceptibility, providing new insights into the pathogenesis of myopia.

## Introduction

Highlights

- Rising myopia concerns: This study acknowledges a significant increase in myopia prevalence globally, spotlighting the yet-to-be-fully-understood aetiology of this condition. It further delves into the less explored territory of the potential link between myopia and inflammation.
- Innovative research approach: Utilizing a bidirectional Mendelian randomization (MR) approach and animal models, this study ambitiously sets out to unravel the causal relationship between inflammation markers and myopia, a novel exploration in the field.
- Extensive data analysis: The research harnesses data from a large-scale genome-wide association study (GWAS) involving over 460 000 European ancestry subjects, providing a robust foundation for the investigation. Employing nine different MR analysis techniques, with inverse variance weighting (IVW) taking the lead, showcases the study’s comprehensive analytical methodology.
- Significant findings: The study unveils that elevated levels of IL-2 and IL-2ra are significantly associated with an increased risk of myopia, while lower levels of C-reactive protein (CRP) and tumour necrosis factor alpha (TNF-α) are linked to a heightened myopia risk, offering new insights into the inflammation-myopia nexus.
- Experimental validation: Through the examination of IL-2 expression in guinea pigs subjected to form-deprivation myopia (FDM), the study not only corroborates its genetic findings but also enriches the understanding of myopia’s pathogenesis from an experimental perspective.
- Implications for future research and treatment: By establishing a plausible causal link between specific inflammatory markers and myopia, this research opens new avenues for future studies and potentially, for the development of targeted therapeutic interventions for myopia prevention and management.

Myopia, a prevalent ocular disorder characterized by eye axis elongation, primarily develops during childhood, leading to blurred vision. It has become a global public health issue, with projections suggesting nearly 50% of the world’s population, or 4.758 billion individuals, will be affected by 2050, including 938 million with high myopia^1,2^. Notably, in Taiwan, high myopia prevalence reaches 21.2% among those aged 18–40. High myopia can lead to severe ocular diseases, such as retinal detachment and glaucoma, causing irreversible visual damage^3^. Current research suggests that the causes of myopia are mainly a combination of genetic and environmental factors. With the improvement of the level of social education, the extension of close work time, and the reduction of outdoor activity time, the incidence of myopia shows a trend of becoming younger, more widespread, and more severe. Currently, there are a series of methods and means for the management and treatment of myopia, such as low-concentration atropine, low-intensity red light, orthokeratology lenses, etc^4^. However, the key to controlling the rapid rise in the global incidence of myopia is to understand the causes and mechanisms of the onset and development of myopia.

The causes and mechanisms of myopia development are not fully elucidated, and management of myopia has not been effectively prevented or intervened from an aetiological perspective. Recent studies have found a close association between the development of myopia and immune inflammation. The study found that the prevalence of myopia is higher among patients with allergic conjunctivitis compared to patients without allergic conjunctivitis^5^. Abnormal changes in various inflammatory factors have been detected in the ocular fluid of mice with simple myopia, and the use of cyclosporine can downregulate the expression of inflammatory factors, thereby inhibiting the progression of myopia^5,6^. In patients with high myopia, a positive correlation was found between the level of IL-6 in the aqueous humour and the length of the eye axis, and using inhibitors of IL-6 could inhibit the progression of myopia^7^. These observational studies all prove that inflammation is a risk factor for the occurrence of myopia, playing an important role in the development process of myopia. However, the conclusions of these observational studies may be affected by various confounding factors, leading to a decrease in the credibility of the results and an inability to prove a causal relationship between inflammation and myopia.

Mendelian randomization (MR) is a method that uses genetic variants as instrumental variables to circumvent the effects of confounding factors, thereby inferring the causal relationship between exposure and outcomes. Genetic variants are acquired at conception and randomly distributed, unaffected by the environment or lifestyle. MR studies have been applied to a variety of diseases. A two-sample MR study found that C-reactive protein (CRP) has a protective effect against schizophrenia, and an increase in IL-1Ra levels could increase the risk of developing schizophrenia^8^. Similarly, Mendelian studies on Parkinson’s disease discovered that the pro-inflammatory activity of IL-6 might be a decisive factor in its development^9^. These findings play an important role in the treatment and intervention of diseases.

According to current literature, there have been no studies found that use the MR method to assess the causal relationship between inflammation and the occurrence of myopia. In this study, We used 27 known and common inflammatory factors as exposures and studied their association with myopia using information from different genome-wide association study (GWAS) data sources. Furthermore, we explored their causal relationship and analyzed the reliability of the results through MR, finally verifying it in experimental myopic animals. The results of this study may provide strong evidence for potential targets in the prevention and treatment of myopia.

## Methods

### Study design and date sources

**Figure 1:**
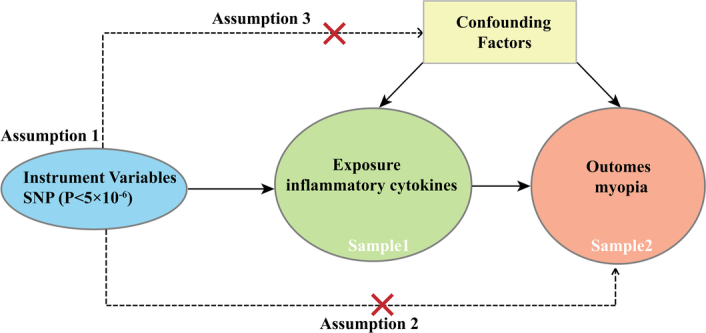
Schematic of the study design for the two-sample Mendelian randomization analysis. Twenty-seven common inflammatory factors were selected as the exposure and myopia as the outcome to explore the relationship between the 27 common inflammatory factors and myopia. The loop diagram demonstrates the three basic Mendelian assumptions of correlation, independence, and exclusion.

Leveraging a two-sample MR analysis model, we employed summary genetic data from separate samples to evaluate the causal influence of circulating inflammatory cytokines on myopia. A schematic representation of the overall MR approach is illustrated in Fig. 1. We identified the following inflammation biomarkers for examination as exposures: CRP, IL-1ra, IL-2ra, IL-2, IL-6, IL-7, IL-8, IL-10, IL-13, IL-14, IL-16, IL-17, IL-18, monocyte chemotactic protein 1 (MCP-1), Macrophage migration inhibitory factor (MIF), monokine production induced by interferon gamma (MIG), macrophage inflammatory protein-1a (MIP1a), MIP1b, cutaneous T-cell-attracting chemokine (CTACK), Eotaxin, growth-regulated oncogene-a (GROa), tumour necrosis factor alpha (TNF-α), TNF-b, RANTES, TNF-related apoptosis-inducing ligand (TRAIL), chemokine (C-X-C motif) ligand 9 (CXCL9). MR analysis models were then applied to determine the causal impacts of these 27 circulating inflammatory cytokines on myopia.

We sourced published genetic variants associated with circulating inflammatory cytokines from the most exhaustive cytokine-related GWAS meta-analysis to date, encompassing three independent cohorts: the Cardiovascular Risk in Young Finns Study (YFS), FINRISK 1997, and FINRISK 2002, involving up to 8293 Finnish participants in total. The primary outcome of this investigation was the lifetime risk of developing myopia. The summary statistics for myopia were obtained from the most extensive GWAS published to date (ID: ukb-b-6353, available at https://gwas.mrcieu.ac.uk/datasets/ukb-b-6353/). This GWAS on myopia examined the relationship between up to 9 851 867 genotyped single-nucleotide polymorphisms (SNPs) and myopia, including data from 460 536 individuals of European descent, comprising 37 362 patients with myopia and 423 174 control individuals.

### Selection of SNPs

We conducted a search for the IV SNPs related to the outcome of interest, myopia, within large-scale GWAS data (ID: ukb-b-6353, accessible via https://gwas.mrcieu.ac.uk/datasets/ukb-b-6353/). The dataset pertaining to myopia was specifically sourced from ukb-b-6353. All studies that contributed data to these GWAS meta-analyses had previously obtained ethical approval from the appropriate institutional review boards. In our analysis, we exclusively utilized summarized data from these studies, negating the necessity for additional ethics approval. Comprehensive information regarding all SNPs utilized in this research can be found in the Supplementary materials, Supplemental Digital Content 2, http://links.lww.com/MS9/A541.

### Animals

The sample size was calculated according to the formula *n*=(2*σ^2^*(*Z*_α/2_+*Z*_β_) ^2^)/δ^2^ ^10^. Sixty male tricolour, British short-haired breed, healthy guinea pigs, aged 2 weeks, special pathogen free (SPF), and weighing between 100 and 120 g were acquired from Jiangsu Danyang Changyi Laboratory Animal Breeding Co. Throughout the study, all guinea pigs were maintained in a controlled environment: a room temperature of 25 °C ± 1 °C, humidity at 55% ± 5%, and subjected to alternating 12-h light-dark cycles (300 lux from 8:00 a.m. to 8:00 p.m., 0 lux from 8:00 p.m. to 8:00 a.m.), with constant ambient noise kept at 30 dB ± 5% and daily ventilation ensured. They were provided with uniform water and feed, including hay or green fodder once daily in addition to pellet feed, enriched with crude fibre and vitamin C (fresh vegetables) in consistent amounts each day. The exclusion criteria for the study were established as follows: (1) refractive interstitial opacity (including corneal foreign body, lens opacity, vitreous clouding, etc.); (2) congenital myopia; (3) refractive error greater than 2 dioptre (D); (4) congenital developmental anomalies; (5) primary refractive error greater than 6D; and 6. evidence of infection, injuries, or death during the course of the experiment.

All animals were accommodated within the Department of Animal Experimentation at the Shanghai Public Health Clinical Center, where they were provided with standardized and uniform care. All animal experiments were conducted in compliance with the regulations set forth by the Association for Research in Vision and Ophthalmology (ARVO) regarding the use of laboratory animals in ophthalmic and vision research. All of our animal experiments were reported in full compliance with ARRIVE criteria^11^, Supplemental Digital Content 1, http://links.lww.com/MS9/A540. All studies were approved by the ethics Committee of Jinshan Hospital of Fudan University.

### Animal grouping and model establishment

The 32 guinea pigs were equally and randomly allocated into two groups, with each group having the right eye designated as the experimental eye for induced myopia. To ensure the right eye was occluded while permitting free blinking, the guinea pigs were fitted with a semi-transparent latex balloon mask over their heads. Care was taken to expose the ears, nose, and mouth fully, thereby not inhibiting their natural activities. The integrity of the eye masks was checked daily, and any that had fallen off, suffered damage, were contaminated, or had been displaced were immediately replaced.

### Ocular refraction and axial length measurement

Refractive measurements for both eyes of all animals were taken at 0W and after 4 weeks (4W) using a small animal photorefractor (SriaTech Company, Germany) set at the following parameters: wavelength: 875 nm; frame rate: greater than 115 Hz; working distance: 56 cm. Before each measurement, pupils were dilated using a compound tropicamide eye drop (Santen Pharmaceutical Co., Ltd.) for 10 minutes. All measurements were conducted in a dark room, and the average value of the refractive error was recorded and saved once it stabilized.

Following the application of corneal surface anaesthesia with Oxybuprocaine hydrochloride eye drops (Santen Pharmaceutical Co.), the axial length (AL) of the guinea pigs’ eyes was measured using ophthalmic A-ultrasound equipment (Suzhou 66 Optical Science and Technology Co., Ltd.). The A-ultrasound probe was carefully positioned perpendicular to the centre of the cornea and in gentle contact to avoid exerting pressure on the corneal surface. Images and readings were taken only when the images appeared clear and stable. The final result was derived from the average of three measurements, each with an accuracy of 0.01 mm. Refraction and axial length were measured by a professional optometrist who was unaware of the experimental group assignments.

### Enzyme-linked immunosorbent assay

Following 4 weeks of myopia induction, vitreous samples were collected from 16 successfully modelled guinea pigs and 16 controls. Animals were euthanized using an overdose of 1% sodium pentobarbital administered intraperitoneally. The right eyes were enucleated, and the vitreous tissues were immediately cryopreserved in liquid nitrogen. For the enzyme-linked immunosorbent assay (ELISA), we utilized the guinea pig white IL-2 ELISA kit (Jiangsu Meimian Industrial Co., Ltd). We plotted standard concentrations against OD values on graph paper to derive a standard curve, from which we determined sample concentrations and calculated dilution factors for target concentrations.

### Statistical analysis

In this study, we employed nine MR analysis methods: inverse-variance weighted (IVW) with both fixed and random effects, simple median, weighted median, penalized weighted median, simple mode, weighted mode, and MR-Egger. We primarily used the random-effects IVW method to analyze the results, as it offers robust causal estimates, especially in the presence of heterogeneity. The statistical criteria for robust instrumental variables included an F-value greater than 10 and R^2^ less than 0.1, with the F-value and R^2^ calculated in accordance with the methodology of Chen *et al.* ^12^.

Due to the IVW method’s assumption that all instrumental variables must adhere to MR principles, we conducted additional sensitivity analyses using the weighted median estimator and MR-Egger. The weighted median estimator can provide a consistent causal estimate when over half of the instrumental variables are valid. In contrast, the MR-Egger estimate remains unbiased, assuming that the instrument strength is independent of pleiotropic effects. Furthermore, we used IVW methods with MR-Egger intercept and Cochran’s Q statistics to assess the pleiotropy and heterogeneity of individual SNPs. As long as the intercept did not significantly differ from 0 (*P*>0.05), pleiotropic effects were considered absent. The value of Cochrane’s Q was used to evaluate heterogeneity.

Additionally, MR-Egger regression was performed to detect and correct for pleiotropy, assess causal effects, and determine if directional horizontal pleiotropy affected the outcomes. A leave-one-out analysis was executed to examine the MR analysis results’ stability, pinpointing any individual SNP outlier’s impact. Outlier SNPs and those associated with myopia identified by MR-PRESSO were removed to refine the analysis further.

Building on the findings of the prior research, a causal relationship was considered statistically significant under three specific criteria: The *P* value of IVW less than 0.05, there is consistency in the direction of estimates among the IVW, MR-Egger, and weighted median methods, and the MR-Egger intercept test’s *P* value exceeds 0.05. All statistical analyses were undertaken using the “Two Sample MR” package in R version 3.4.1 (R Foundation for Statistical Computing), and a two-tailed *P* value less than 0.05 was considered indicative of statistical significance.

Animal experimental data were statistically evaluated using SPSS 25.0 software (IBM Corp.), with outcomes presented as means ± standard deviations. For statistical analyses between groups, independent sample *t*-tests was employed, depending on the nature of the data and the comparisons being made.

## Results

### Principal MR results

We identified SNPs linked to these circulating inflammatory cytokines to serve as instrumental variables (IVs). Initially, we established a genome-wide significance threshold of *P* less than 5×10^−8^ to select SNPs strongly associated with both myopia and inflammatory cytokines. When this criterion resulted in only a minimal number of SNPs, we adopted a more lenient cutoff (*P*<5×10^−6^). Furthermore, to mitigate the effects of linkage disequilibrium (LD), we stipulated criteria (LD; r^2^ <0.001, LD distance >10 000 kb). Upon setting the genome-wide significance threshold, only CRP yielded three or more valid genetic variants among the 27 evaluated inflammatory factors. Hence, we applied a relaxed threshold (*P*<5 × 10^−6^) to ensure an adequate number of SNPs for further analysis. This approach allowed for a comprehensive evaluation of 27 inflammatory factors as instrumental variables, including CRP, IL-1ra, IL-2ra, IL-2, IL-6, IL-7, IL-8, IL-10, IL-13, IL-14, IL-16, IL-17, IL-18, MCP-1, MIF, MIG, MIP1a, MIP1b, CTACK, eotaxin, GROa, RANTES, TRAIL, and CXCL9. The SNP count for each factor was as follows: CRP with 296 SNPs, IL-1ra with 6 SNPs, and so on, with each factor’s SNP counts detailed accordingly (Table 1, Table S5, Supplemental Digital Content 2, http://links.lww.com/MS9/A541). Importantly, all included SNPs had strong F-statistics greater than 10 and R² values less than 0.1, confirming their strong association with the respective exposure variable.

**Table 1:** Twenty-seven causal relationship between inflammatory factors and myopia.

Phenotype	N SNPs	Methods	OR 95% CI	*P*	Cochran’s Q	*P*	MR intercept	*P*	MR-PRESSO
CRP	296	IVW (multiplicative random effects)	0.996(0.994–0.999)	0.002	426.755	7.75E-07	0.0003	0.0006	<0.001
		Weighted median	0.993(0.990–0.996)	4.05E-06	NA	NA			
		MR-Egger	0.992(0.988–0.995)	3.21E-06	409.829	8.94E-06			
IL-2	10	IVW (multiplicative random effects)	1.003(1.001–1.005)	0.001	3.748	0.927	−8.56E-05	0.854	0.904
		Weighted median	1.003(1.000–1.007)	0.079	NA	NA			
		MR-Egger	1.003(0.988–1.009)	0.258	3.712	0.882			
IL-6	7	IVW (multiplicative random effects)	0.999(0.993–1.005)	0.651	10.424	0.108	−0.001	0.105	0.137
		Weighted median	1.001(0.995–1.008)	0.661	NA	NA			
		MR-Egger	1.006(0.997–1.016)	0.226	5.851	0.321			
IL-7	11	IVW (multiplicative random effects)	1.001(0.999–1.003)	0.208	7.603	0.668	−5.63E-05	0.936	0.745
		Weighted median	1.001(0.999–1.004)	0.310	NA	NA			
		MR-Egger	1.001(0.996–1.006)	0.602	7.596	0.575			
IL-8	4	IVW (multiplicative random effects)	1.001(0.998–1.004)	0.633	1.994	0.574	0.0003	0.578	0.67
		Weighted median	1.000(0.996–1.004)	0.965	NA	NA			
		MR-Egger	0.999(0.993–1.005)	0.795	1.561	0.458			
IL-10	22	IVW (multiplicative random effects)	1.000(0.998–1.002)	0.910	23.725	0.307	−0.0003	0.335	0.293
		Weighted median	1.002(0.999–1.005)	0.176	NA	NA			
		MR-Egger	1.002(0.998–1.006)	0.374	22.621	0.308			
IL-13	9	IVW (multiplicative random effects)	1.001(1.000–1.002)	0.163	4.133	0.845	−0.0003	0.556	0.888
		Weighted median	1.001(0.999–1.004)	0.259	NA	NA			
		MR-Egger	1.002(0.998–1.005)	0.313	3.751	0.808			
IL-14	11	IVW (multiplicative random effects)	1.003(0.999–1.007)	0.124	10.759	0.377	−5.88E-05	0.913	0.385
		Weighted median	1.002(0.997–1.007)	0.504	NA	NA			
		MR-Egger	1.003(0.997–1.010)	0.372	10.743	0.294			
IL-16	10	IVW (multiplicative random effects)	1.003(0.999–1.007)	0.327	9.023	0.435	−0.0003	0.521	0.512
		Weighted median	0.999(0.997–1.001)	0.849	NA	NA			
		MR-Egger	1.000(0.997–1.003)	0.948	8.542	0.382			
IL-17	10	IVW (multiplicative random effects)	0.999(0.995–1.004)	0.792	11.235	0.260	−0.0003	0.712	0.745
		Weighted median	0.999(0.994–1.004)	0.747	NA	NA			
		MR-Egger	1.001(0.991–1.011)	0.833	11.033	0.200			
IL-18	13	IVW (multiplicative random effects)	1.001(0.998–1.003)	0.694	22.636	0.031	0.0002	0.642	0.67
		Weighted median	1.001(0.998–1.004)	0.569	NA	NA			
		MR-Egger	0.999(0.994–1.005)	0.844	22.176	0.023			
IL-1ra	6	IVW (multiplicative random effects)	1.000(0.998–1.002)	0.975	1.792	0.877	0.0009	0.393	0.884
		Weighted median	0.999(0.995–1.004)	0.801	NA	NA			
		MR-Egger	0.995(0.985–1.006)	0.419	0.876	0.928			
IL-2ra	7	IVW (multiplicative random effects)	1.002(1.000–1.003)	0.049	3.285	0.772	5.83E-06	0.993	0.709
		Weighted median	1.001(0.998–1.003)	0.647	NA	NA			
		MR-Egger	1.002(0.998–1.005)	0.406	3.285	0.656			
CTACK	10	IVW (multiplicative random effects)	0.999(0.997–1.008)	0.589	8.449	0.391	−0.001	0.137	0.297
		Weighted median	1.000(0.997–1.003)	0.991	NA	NA			
		MR-Egger	1.003(0.998–1.008)	0.280	11.329	0.254			
CXCL9	12	IVW (multiplicative random effects)	0.999(0.996–1.002)	0.402	16.862	0.112	0.002	0.059	0.141
		Weighted median	0.999(0.995–1.002)	0.421	NA	NA			
		MR-Egger	0.994(0.989–0.999)	0.043	11.611	0.312			
Eotaxin	16	IVW (multiplicative random effects)	0.998(0.994–1.001)	0.164	23.622	0.072	−0.0007	0.264	0.079
		Weighted median	0.999(0.994–1.003)	0.532	NA	NA			
		MR-Egger	1.002(0.994–1.011)	0.617	21.539	0.089			
GROa	8	IVW (multiplicative random effects)	0.999(0.997–1.000)	0.132	4.974	0.663	5.85E-05	0.939	0.731
		Weighted median	0.999(0.997–1.001)	0.482	NA	NA			
		MR-Egger	0.999(0.995–1.003)	0.558	4.967	0.548			
IFNg	11	IVW (multiplicative random effects)	1.000(0.996–1.005)	0.848	13.815	0.182	0.0008	0.240	0.237
		Weighted median	0.999(0.993–1.004)	0.594	NA	NA			
		MR-Egger	0.996(0.988–1.004)	0.362	11.745	0.228			
MCP-1	13	IVW (multiplicative random effects)	1.000(0.997–1.004)	0.856	18.511	0.101	−0.0003	0.535	0.142
		Weighted median	0.999(0.995–1.003)	0.660	NA	NA			
		MR-Egger	1.003(0.995–1.010)	0.531	17.844	0.085			
MIP1a	7	IVW (multiplicative random effects)	1.004(1.000–1.009)	0.065	8.578	0.199	0.001	0.361	0.234
		Weighted median	1.003(0.998–1.008)	0.288	NA	NA			
		MR-Egger	0.997(0.984–1.012)	0.737	7.134	0.211			
MIP1b	20	IVW (multiplicative random effects)	1.000(0.998–1.002)	0.996	23.756	0.206	0.0009	0.009	0.185
		Weighted median	0.999(0.996–1.001)	0.221	NA	NA			
		MR-Egger	0.997(0.995–1.000)	0.043	15.316	0.640			
MIG	12	IVW (multiplicative random effects)	0.999(0.996–1.002)	0.402	16.862	0.112	0.002	0.059	0.146
		Weighted median	0.999(0.995–1.002)	0.403	NA	NA			
		MR-Egger	0.994(0.989–0.999)	0.043	11.611	0.312			
MIF	6	IVW (multiplicative random effects)	0.997(0.994–1.001)	0.119	4.058	0.541	−0.0003	0.651	0.582
		Weighted median	0.997(0.992–1.002)	0.201	NA	NA			
		MR-Egger	0.999(0.993–1.005)	0.708	3.819	0.431			
RANTES	10	IVW (multiplicative random effects)	1.000(0.997–1.004)	0.877	11.934	0.217	0.0008	0.394	0.211
		Weighted median	1.001(0.997–1.005)	0.698	NA	NA			
		MR-Egger	0.996(0.987–1.006)	0.454	10.834	0.211			
TNF-α	3	IVW (multiplicative random effects)	0.995(0.994–0.996)	<0.001	0.105	0.949	7.38E-05	0.949	NA
		Weighted median	0.995(0.990–1.001)	0.082	NA	NA			
		MR-Egger	0.995(0.985–1.005)	0.510	0.099	0.753			
TNF-b	4	IVW (multiplicative random effects)	1.000(0.998–1.001)	0.817	1.492	0.684	0.0003	0.644	0.643
		Weighted median	1.001(0.998–1.002)	0.730	NA	NA			
		MR-Egger	0.999(0.996–1.002)	0.669	1.203	0.548			
TRAIL	18	IVW (multiplicative random effects)	1.000(0.990–1.002)	0.526	13.572	0.697	0.0001	0.734	0.747
		Weighted median	1.001(0.998–1.003)	0.546	NA	NA			
		MR-Egger	1.000(0.998–1.002)	0.824	13.452	0.639			

CRP, C-reactive protein; CTACK, cutaneous T-cell-attracting chemokine; CXCL9,chemokine (C-X-C motif) ligand 9; GROa, growth-regulated oncogene-a; IFNg, interferon gamma; IL, interleukin; MCP-1, monocyte chemotactic protein 1; MCP3, monocyte-specific chemokine 3; MIF, macrophage migration inhibitory factor; MIG, monokine induced by interferon gamma; MIP1a, macrophage inflammatory protein-1a; MIP1b, macrophage inflammatory protein-1b; MR, Mendelian randomization; MR-PRESSO, MR Pleiotropy RESidual Sum and Outlier; NA, not applicable; OR, odds ratio; SNP, single-nucleotide polymorphisms; TNF-a, tumour necrosis factor alpha; TNF-b, tumour necrosis factor beta; TRAIL, TNF-related apoptosis-inducing ligand.

**Figure 2:**
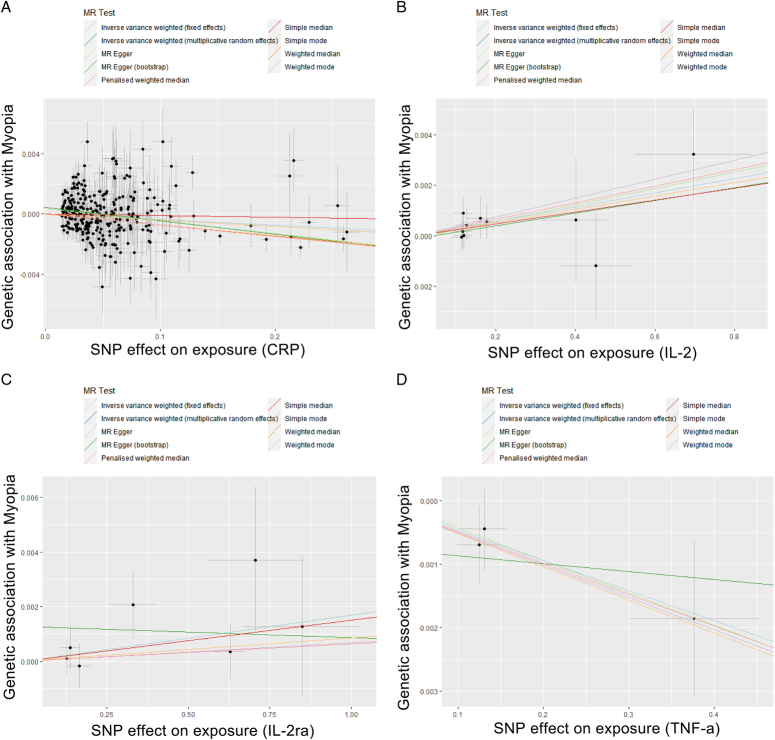
Scatter plot of SNPs associated with inflammatory factors and their risk of myopia. (A) The association of each SNP with CRP and their risk of myopia. (B) The association of each SNP with IL-2 and their risk of myopia. (C) The association of each SNP with IL-2ra and their risk of myopia. (D) The association of each SNP with TNF-α and their risk of myopia. CRP, C-reactive protein; IL, interleukin; MR, Mendelian randomization; SNP, single-nucleotide polymorphisms; TNF-α, tumour necrosis factor alpha.

The primary findings from the MR analysis of the 27 common inflammatory factors are thoroughly outlined in Table 1. Noteworthy findings include significant associations of IL-2 [IVW odds ratio (OR): 1.003, 95% CI: 1.001–1.005, *P*=0.001], IL-2ra (IVW OR: 1.002, 95% CI: 1.000–1.003, *P*=0.049), CRP (IVW OR: 0.996, 95% CI: 0.994–0.999, *P*=0.002), and TNF-α (IVW OR: 0.995, 95% CI: 0.994–0.996, *P*<0.001) with myopia risk. Concordance in outcomes among the IVW, weighted median, and MR-Egger methods was evident for CRP, IL-2, IL-2ra, and TNF-α, reinforcing the robustness of the associations. Sensitivity analyses indicated no single SNP outliers exerted a disproportionate influence on the causality estimates, as revealed by the leave-one-out approach. Scatter plots detailing the MR analyses for CRP, IL-2, IL-2ra, and TNF-α in relation to myopia are presented in Fig. 2, which depict the MR regression slopes and individual SNP causal estimates. Importantly, the absence of a significant intercept in these plots suggests that no directional pleiotropy was detected. For a comprehensive view, scatterplot results for all 27 inflammatory factors in relation to myopia can be found in the Supplementary Materials S2, Supplemental Digital Content 2, http://links.lww.com/MS9/A541.

### Heterogeneity and pleiotropy analysis

The Supplementary Material S1, Supplemental Digital Content 2, http://links.lww.com/MS9/A541 presents funnel plots, vital for evaluating heterogeneity among genetic variants. These plots are mostly symmetrical, suggesting minimal heterogeneity, with TNF-α as an outlier due to its fewer SNPs. Heterogeneity tests revealed no significant disparities (*P*>0.05) for most inflammatory factors, except CRP and IL-18, as determined by both the IVW and MR-Egger methods. Additionally, horizontal pleiotropy analysis indicated no significant pleiotropy (*P*>0.05) for CRP and MIP1b, contrasting with other factors where pleiotropy was suggested (*P*<0.05), as summarized in Table 1.

**Figure 3:**
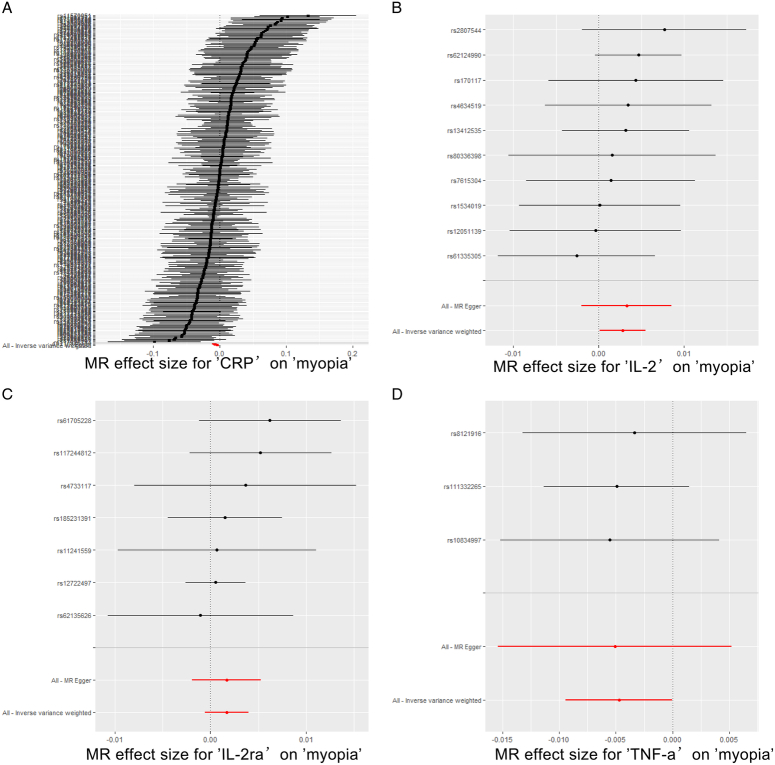
Forest plot of SNPs associated with inflammatory factors and their risk of myopia. (A) Forest plot of SNPs associated with CRP and the risk of myopia. (B) Forest plot of SNPs associated with IL-2 and the risk of myopia. (C) Forest plot of SNPs associated with IL-2ra and the risk of myopia. (D) Forest plot of SNPs associated with TNF-α and the risk of myopia. CRP, C-reactive protein; IL, interleukin; MR, Mendelian randomization; SNP, single-nucleotide polymorphisms; TNF-α, tumour necrosis factor alpha.

**Figure 4:**
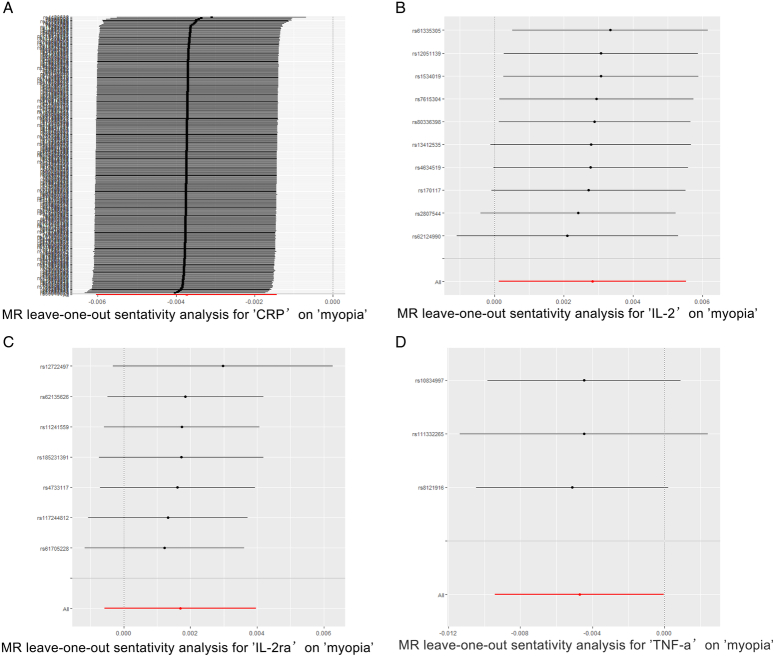
Leave-one-out of SNPs associated with inflammatory factors. (A) Leave-one-out of SNPs associated with CRP and the risk of myopia. (B) Leave-one-out of SNPs associated with IL-2 and the risk of myopia. (C) Leave-one-out of SNPs associated with IL-2ra and the risk of myopia. (D) Leave-one-out of SNPs associated with TNF-α and the risk of myopia. CRP, C-reactive protein; IL, interleukin; MR, Mendelian randomization; SNP, single-nucleotide polymorphisms; TNF-α, tumour necrosis factor alpha.

To enhance confidence in our results’ robustness, we employed a Leave-One-Out and Forest plot analysis, as detailed in Supplementary Material S3, Supplemental Digital Content 2, http://links.lww.com/MS9/A541 and illustrated in Fig. 3. This approach entailed sequentially removing each SNP to assess its influence on the overall effect of inflammatory factors on myopia risk. The outcomes, depicted in Fig. 4, confirm that no individual SNP disproportionately affected the results, affirming the stability and reliability of our findings. Furthermore, forest plot outcomes for all 27 inflammatory factors in relation to myopia are provided in Supplementary Materials S4, Supplemental Digital Content 2, http://links.lww.com/MS9/A541, offering a comprehensive view of the effect sizes and confidence intervals, thereby reinforcing the integrity of our analysis.

### Alterations in refractive and axial length in the form-deprivation myopia (FDM) guinea pig group

**Figure 5:**
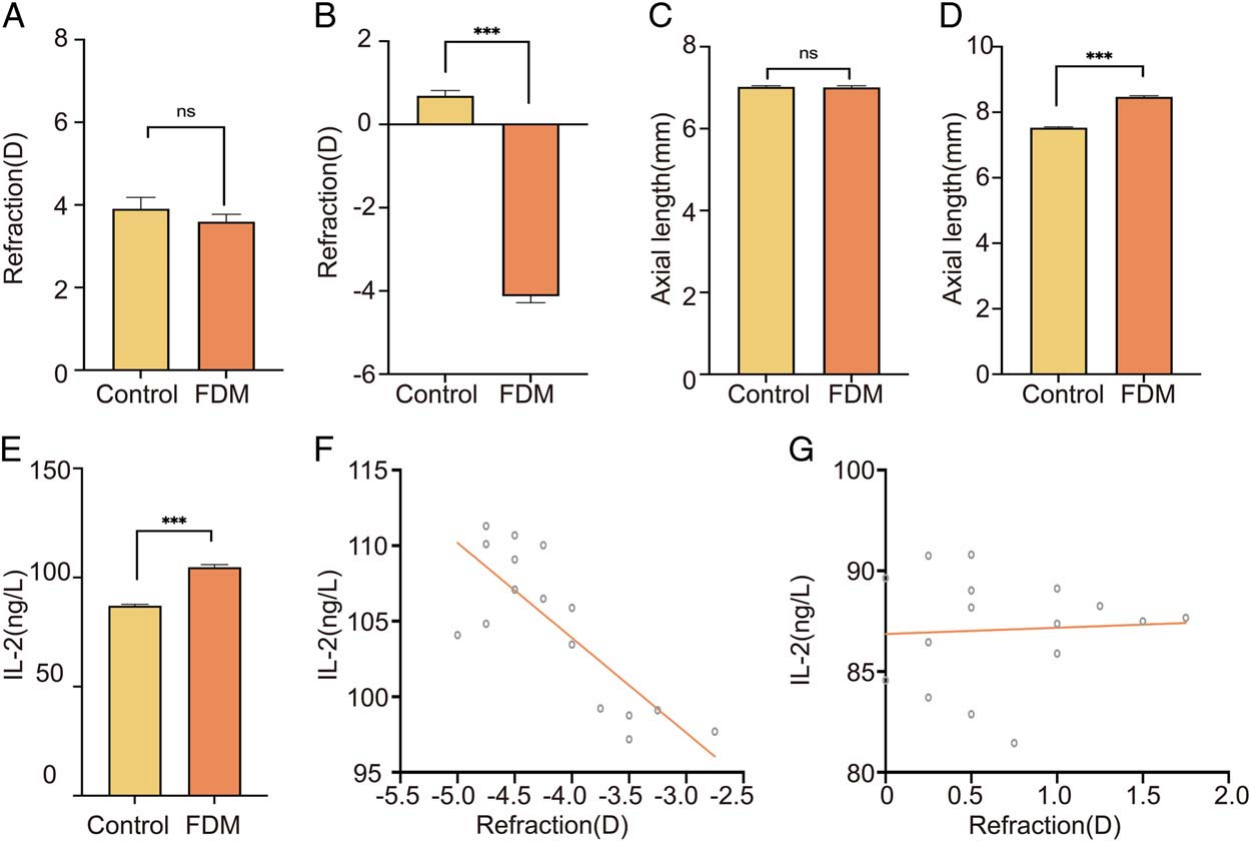
Changes in intravitreal IL-2 concentration in the right eyes of FDM guinea pigs. (A) Refractive error measurements of the right eyes of the two groups of guinea pigs before modelling (0W) showed no significant difference in refractive error (*P*>0.05). (B) Refraction of the right eyes of guinea pigs in the FDM group after 4W of modelling progressed toward myopia, which was significantly different from that of the control group (*P*<0.001). (C) Axial measurements of the right eyes of the two groups of guinea pigs before modelling (0W) showed no significant difference in axial length (*P*>0.05). (D) The right eye axes of guinea pigs in the FDM group were significantly longer after 4W of modelling, which was significantly different from the control group (*P*<0.001). (E) The intravitreal IL-2 concentration in the right eye of guinea pigs in the FDM group after 4W of modelling was significantly higher than that in the control group (*P*<0.001). (F) Positive correlation between intravitreal IL-2 concentration and myopic refraction in the right eyes of guinea pigs in the FDM group after 4W of modelling (*P*<0.001). (G) There was no significant correlation between intravitreal IL-2 concentration and refractive error in the right eyes of guinea pigs in the control group after 4W of modelling (*P*>0.05). IL, interleukin; FDM, form-deprivation myopia.

Before the myopia induction (0 week), no significant differences were detected in spherical equivalent (SE) and AL between the control group’s right eyes (*n*=16) and those of the FDM group (*n*=16, *P*>0.05), as shown in Fig. 5A, C. However, after the 4-week induction period, significant differences emerged. The SE in the FDM group significantly decreased relative to the control group (*P*<0.001), depicted in Fig. 5B, and the AL in the FDM group significantly increased when compared to the control group (*P*<0.001), also illustrated in Fig. 5D.

### Alterations in the concentration of IL-2 in guinea pig vitreous humour

Following the 4-week induction period, there was a significant increase in intravitreal IL-2 concentration in the right eye of the FDM group (*n*=16) compared to the control group (*n*=16) (*P*<0.001), as illustrated in Fig. 5E. Additionally, within the FDM group, a significant positive correlation was found between IL-2 concentration and the degree of myopic refraction (*P*<0.001), shown in Fig. 5F. Conversely, in the control group, no significant correlation was observed between intravitreal IL-2 concentration and refractive error (*P*>0.05), as depicted in Fig. 5G.

## Discussions

In this two-sample MR study, we explored the causal relationship between exposure to 27 inflammatory cytokines and the development of myopia, identifying a potential pathogenic association with IL-2 and IL-2ra. Our findings suggest that elevated levels of IL-2 and IL-2ra may increase the risk of developing myopia, offering new insights into the pathogenesis of myopia.

In recent years, research has revealed a close association between abnormal expression of inflammatory factors and the occurrence of myopia. Elevated levels of TNF-α and IL-6 have been observed in tree shrews with myopia and patients with high myopia^13,14^. The prevalence of myopia is also higher among patients with both systemic and ocular inflammatory diseases, such as diabetes, systemic lupus erythematosus, and allergic conjunctivitis^7,15^. Moreover, reducing inflammation can slow the progression of myopia^16^. Although existing research suggests a link between myopia and inflammation, observational studies cannot definitively establish a causal relationship between inflammation and myopia. The abnormal expression of inflammatory factors observed in patients with myopia in this study may indicate that low-grade inflammation and immune responses play a pathogenic role in the development of myopia.

In recent years, the pathogenesis of various diseases has been linked to inflammatory factors through MR studies, such as schizophrenia^17^, cancer^18^, Parkinson’s disease^19^, and heart failure^20^. These new discoveries offer fresh insights into the prevention and targeted treatment of these conditions. Our finding intimates that CRP and TNF-α might serve as protective elements against myopia’s evolution. CRP, a nonspecific marker of inflammation, plays a pivotal role in activating the complement system, enhancing phagocytosis, and facilitating the clearance of damaged and necrotic tissues. Contrarily, a study conducted by Wong *et al.* ^21^ revealed no significant distinction in CRP levels between individuals with high myopia and those without, a variation potentially attributable to factors such as the origin of atrial fluid samples (exclusively obtained from patients undergoing cataract surgery), patient age, and demographic diversity. In a cohort study of 58 747 patients with allergic conjunctivitis, it was found that patients with allergic conjunctivitis had a higher prevalence of myopia than those without allergic conjunctivitis, and in the corresponding experimental animal model, TNF-α was found to activate NF-kB and to increase the expression of MMP-2, the latter of which has been shown to promote scleral remodelling in myopia^6^. Although our results indicate a negative correlation between TNF-α and the incidence of myopia, it is crucial to note the limited number of SNPs associated with TNF-α in our analysis. This limitation inherently weakens the reliability of our findings regarding TNF-α, necessitating further research with a more robust dataset to substantiate these observations.

Furthermore, a separate investigation employing a machine learning paradigm to identify risk factors for severe myopia found that CRP did not exacerbate the risk or severity of the condition^22^. Our research delineates a reverse relationship regarding genetically proximate circulating CRP concentrations and myopia. A plausible explanation for this divergence is the potential pleiotropic effects of CRP. Our sensitivity analysis, reflected by a MR intercept with *P* less than 0.05, underscores the presence of horizontal pleiotropic influences when considering CRP as a variable of exposure. This could stem from the inclusion of a substantial number of SNPs, specifically when applying a genome-wide significance threshold of *P* less than 5 × 10^−8^ to identify SNPs closely linked with myopia and inflammatory cytokines, resulting in the selection of 299 SNPs. Despite the scant research on the CRP-myopia nexus and its ambiguous mechanism in myopia’s progression, our consensus across IVW, weighted median, and MR-Egger analyses reaffirms the negative correlation between CRP levels and myopia exposure. By leveraging an expansive dataset, our exploratory study endeavours to shed light on the intricate relationship between CRP and myopia development.

IL-2, a critical cell growth factor, enhances T-cell proliferation and the formation of effector and memory T cells, and has been targeted for therapeutic interventions in tumours, AIDS, and organ transplantation^23^. Its receptor’s α subunit (IL-2Rα) is crucial for sustaining immune tolerance and averting autoimmune reactions. Studies have implicated IL-2 and IL-2Rα in autoimmune uveitis pathogenesis through the modulation of pathogenic Th17 cells, though detailed mechanisms remain under investigation^24^. In allogeneic corneal transplantation, low-dose IL-2 administration may prevent graft rejection by preserving T regulatory (Treg) cells’ suppressive functions through Foxp3 expression enhancement and immunomodulatory cytokine production^25^. IL-2 also promotes retinal pigment epithelium cell migration and extracellular matrix synthesis via the JAK/STAT3 and NF-kB pathways, highlighting its significance in proliferative vitreoretinopathy^26,27^. However, the correlation between IL-2, IL-2Rα, and myopia has been less explored. This study investigates their relationship using genetic variation as an instrumental variable, revealing a positive correlation with myopia development and marking the first observation of increased IL-2 expression in the vitreous of FDM guinea pigs. These findings suggest a potential causal link between IL-2 and IL-2Rα alterations and myopia, opening avenues for further research into their roles in ocular diseases.

Our study is the first to employ MR to examine the causal relationship between 27 common inflammatory markers and the onset of myopia, using large sample size and genetic variants as instrumental variables to reduce confounding factors. However, our research has several limitations. Firstly, our data comes from two extensive GWAS databases without detailed demographic or clinical records of patients, making it impossible to grade the severity of myopia. Secondly, the exclusion of study subjects from European populations could introduce potential bias. Although our study offers new insights into the aetiological treatment and prevention of myopia, future extensive animal studies and clinical trials are required for validation.

## Conclusion

Through MR analysis and inflammation studies in animal models, our research indicates that inflammatory factors serve as a potential aetiology for the development of myopia. In particular, the potential causal link between elevated levels of IL-2 and IL-2ra and increased risk of myopia development. This new insight not only advances our understanding of the multifaceted aetiology of myopia but also offers hope for future prevention and treatment strategies.

## Ethical approval

Ethics approval and consent to participate: This study was approved by the Ethical Committee of Jinshan Hospital of Fudan University (JIEC 2023-S92).

## Consent

The present study followed international, national and/or institutional guidelines for humane animal treatment and complied with relevant legislation. All animals were accommodated within the Department of Animal Experimentation at the Shanghai Public Health Clinical Center, where they were provided with standardized and uniform care. All animal experiments were conducted in compliance with the regulations set forth by the Association for Research in Vision and Ophthalmology (ARVO) regarding the use of laboratory animals in ophthalmic and vision research. All studies were approved by the ethics Committee of Jinshan Hospital of Fudan University.

## Source of funding

This research was financially supported by Shanghai Shenkang Hospital Development Center (No. SHDC2020CR1043B-004), Scientific Research Program of Shanghai Municipal Health Commission(No. 202340282).

## Author contribution

R.L. was mainly responsible for the experimental manipulation and wrote the manuscript, W.S. and T.W. were responsible for the revision and review of the manuscript, H.G. and M.L. were mainly responsible for the statistical analysis, T.L. was responsible for conducting the data review, X.Z. was responsible for the research design and guidance. All authors contributed to this study and approved the final submitted version.

## Conflicts of interest disclosure

The authors affirm that there are no potential conflicts of interest, such as business or financial, in this study.

## Research registration unique identifying number (UIN)

This study does not involve a patient and subject study, so this term is not applicable.

## Guarantor

The guarantor of this study is Xiaodong Zhou.

## Data availability statement

All SNPs included in the study were uploaded to the Supplementary Materials, and all remaining raw data and materials are included in the article and the Supplementary Materials; further inquiries can be directed to the corresponding author.

## Provenance and peer review

This research paper was not invited.

## Supplementary Material
