# Supplementary material for: Causal relationships between inflammatory cytokines and myopia: an analysis of genetic and observational studies
Source: Ann Med Surg (Lond). 2024 Jul 2;86(9):5179–90. doi: 10.1097/MS9.0000000000002325 (PMC11374278; doi:10.1097/MS9.0000000000002325)

**Supplementary Material**

**Contents:**

Supplementary Figure S1.Funnel plot of SNPs associated with inflammatory factors and their risk of myopia.

Supplementary Figure S2.Scatter plot of SNPs associated with inflammatory factors and their risk of myopia.

Supplementary Figure S3.Leave-one-out of SNPs associated with inflammatory factors and their risk of myopia.

Supplementary Figure S4. Forest plot of SNPs associated with inflammatory factors and their risk of myopia.

Supplementary Table S5.All SNPs data in the manuscript.

Consent and ethical clearance copy.


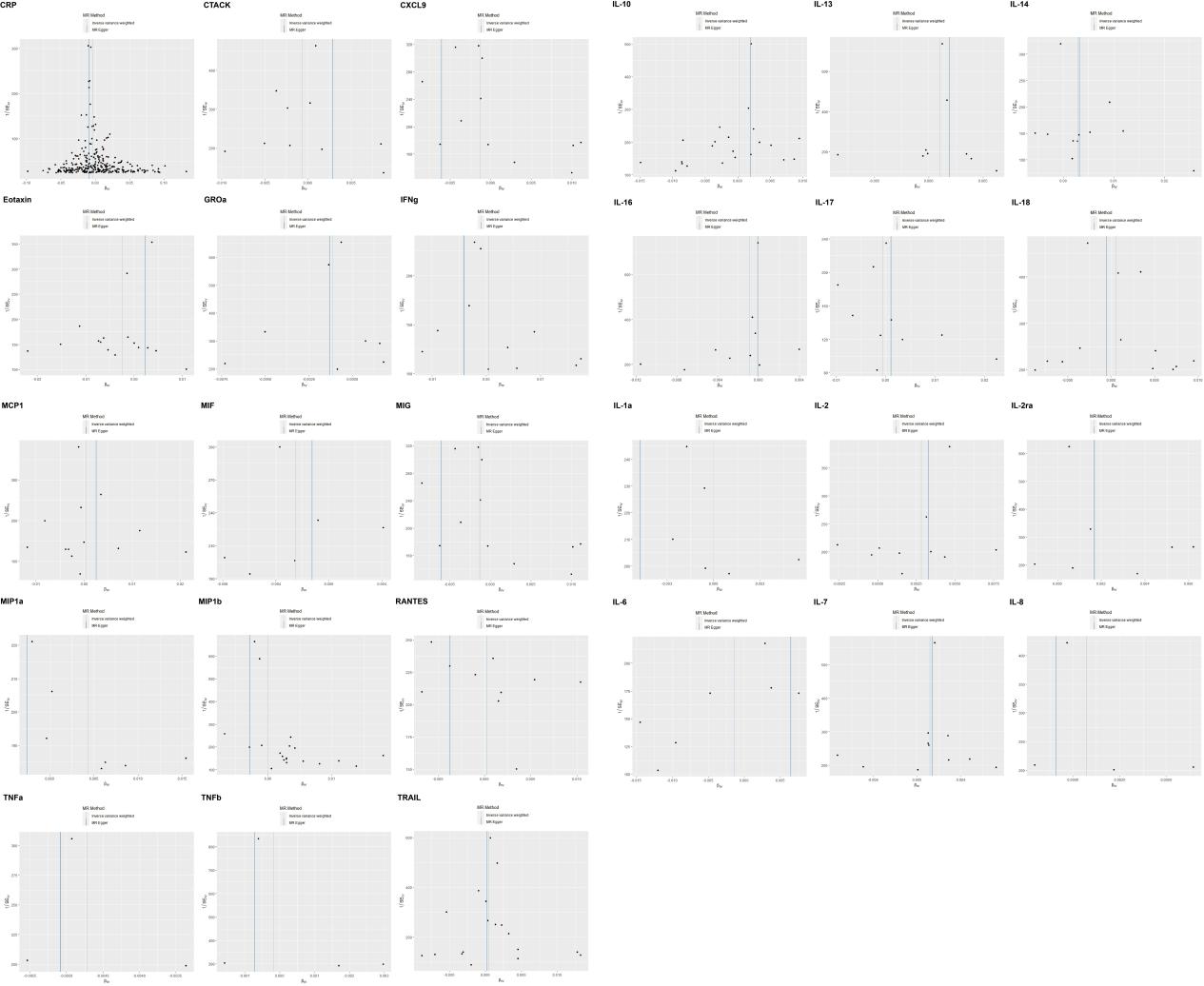


**S1.Funnel plot of SNPs associated with inflammatory factors and their risk of myopia.** The funnel plots show the inverse variance weighted MR estimate of each cytokine single-nucleotide polymorphism with SLE versus 1/standard error (1/SEIV).

Abbreviations:IVW, inversevariance weighted; MR, Mendelian randomization;

MR-PRESSO,MR Pleiotropy RESidual Sum and Outlier;SNP, single-nucleotide polymorphisms;CTACK, cutaneous T cell-attracting chemokine; GROa, growth-regulated oncogene-a; IFNg,interferon gamma;IL, interleukin;MCP1, monocyte chemotactic protein 1; MCP3, monocyte-specific chemokine 3; MIG, monokine induced by interferon gamma;MIP1a, macrophage inflammatory protein-1a; MIP1b, macrophage inflammatory protein-1b; TNFa, tumor necrosis factor alpha; TNFb, tumor necrosis factor beta; TRAIL, TNF-related apoptosis-inducing ligand;CRP,c-reactive protein;MIF,Macrophage migration inhibitory factor;CXCL9,chemokine (C-X-C motif) ligand 9.


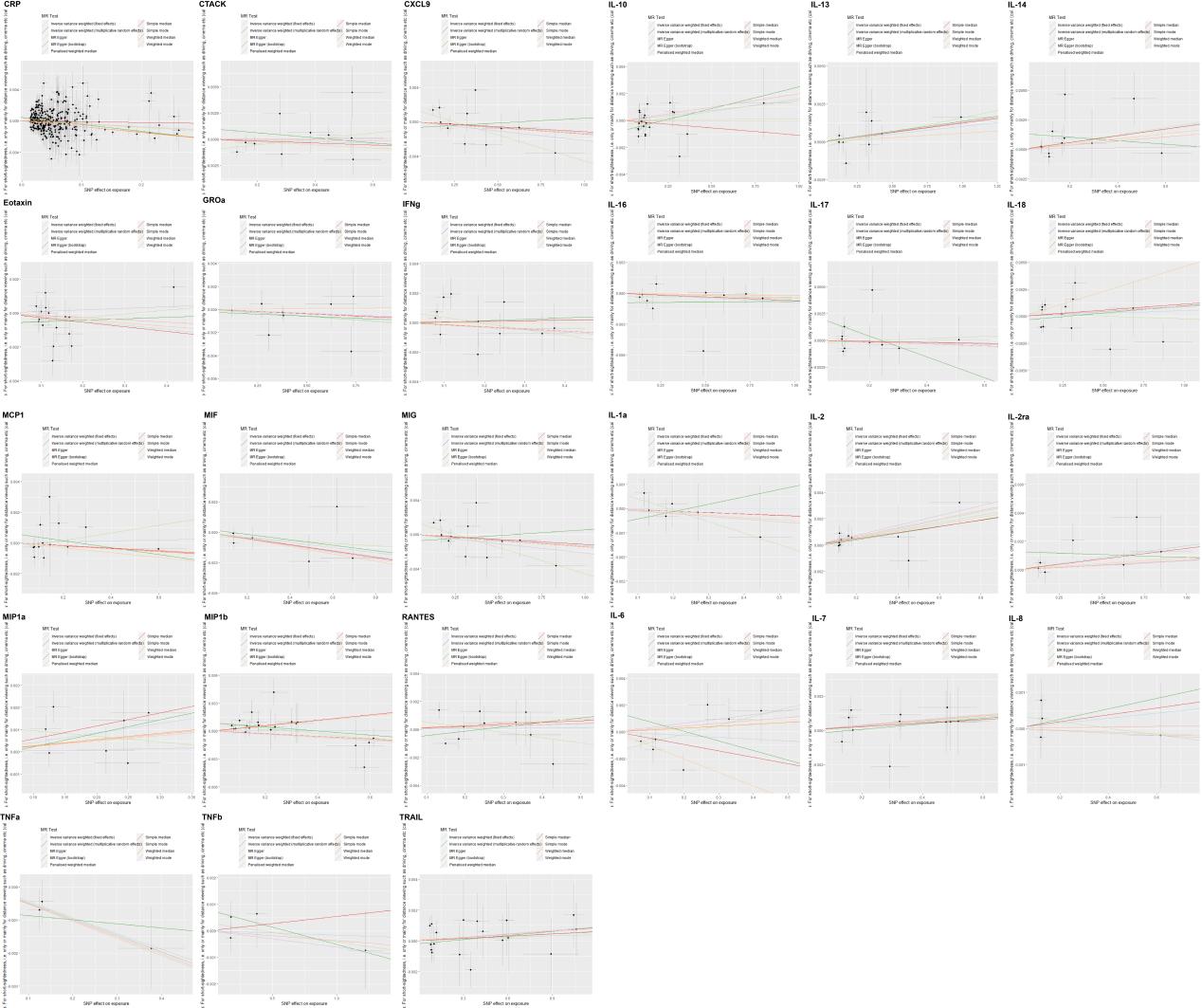


**S2.Scatter plot of SNPs associated with inflammatory factors and their risk of myopia.**Each black dot in the figure represents a genetic variant (SNP), which shows us the association of each SNP with inflammatory factors and myopia. The different colored lines indicate that different simulated fits were used, where the association of each SNP with inflammatory factors directly predicts the association with myopia.

Abbreviations:IVW, inversevariance weighted; MR, Mendelian randomization;

MR-PRESSO,MR Pleiotropy RESidual Sum and Outlier;SNP, single-nucleotide polymorphisms;CTACK, cutaneous T cell-attracting chemokine; GROa, growth-regulated oncogene-a; IFNg,interferon gamma;IL, interleukin;MCP1, monocyte chemotactic protein 1; MCP3, monocyte-specific chemokine 3; MIG, monokine induced by interferon gamma;MIP1a, macrophage inflammatory protein-1a; MIP1b, macrophage inflammatory protein-1b; TNFa, tumor necrosis factor alpha; TNFb, tumor necrosis factor beta; TRAIL, TNF-related apoptosis-inducing ligand;CRP,c-reactive protein;MIF,Macrophage migration inhibitory factor;CXCL9,chemokine (C-X-C motif) ligand 9.


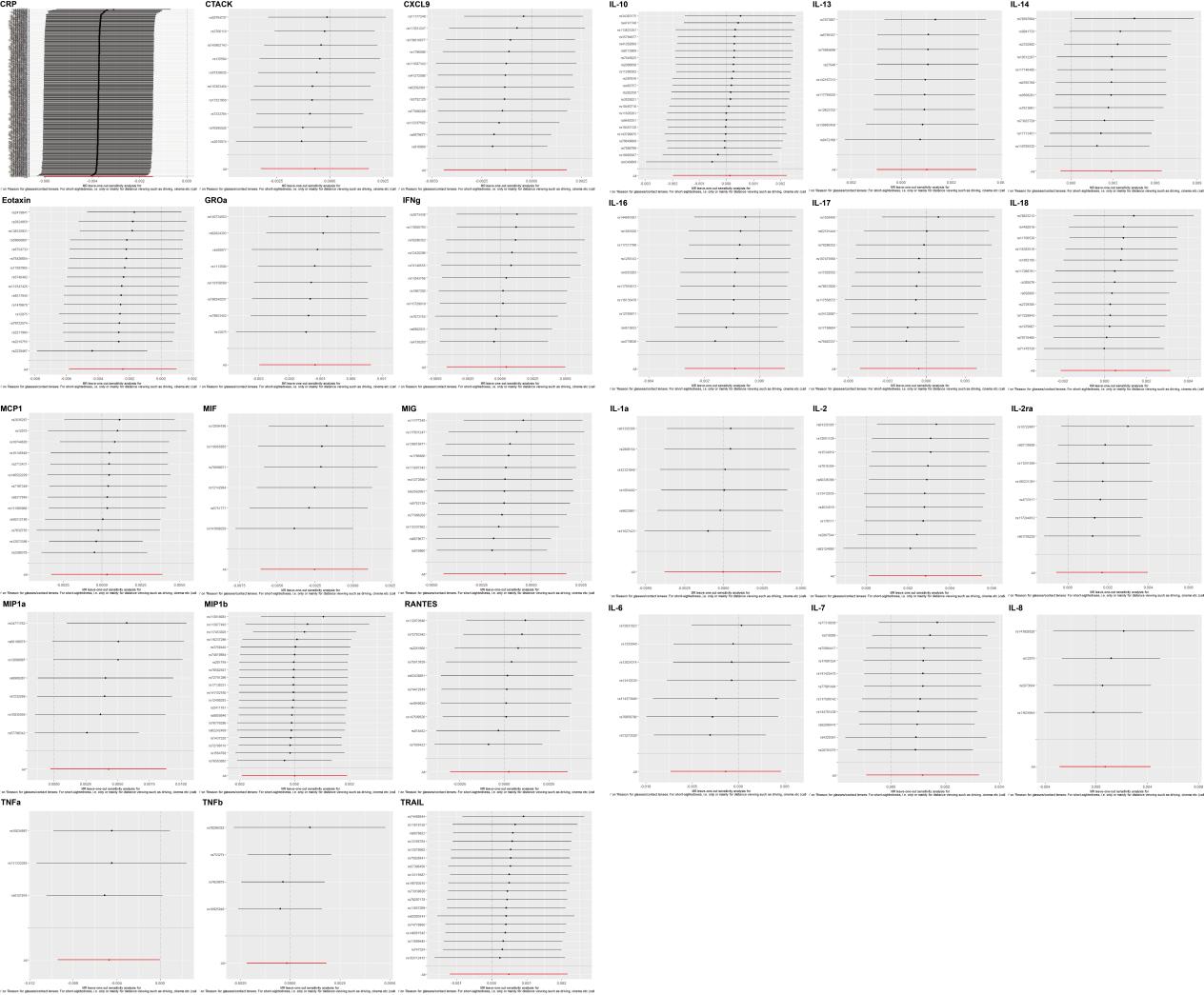


**S3.Leave-one-out of SNPs associated with inflammatory factors and their risk of myopia.**

Abbreviations:IVW, inversevariance weighted; MR, Mendelian randomization;

MR-PRESSO,MR Pleiotropy RESidual Sum and Outlier;SNP, single-nucleotide polymorphisms;CTACK, cutaneous T cell-attracting chemokine; GROa, growth-regulated oncogene-a; IFNg,interferon gamma;IL, interleukin;MCP1, monocyte chemotactic protein 1; MCP3, monocyte-specific chemokine 3; MIG, monokine induced by interferon gamma;MIP1a, macrophage inflammatory protein-1a; MIP1b, macrophage inflammatory protein-1b; TNFa, tumor necrosis factor alpha; TNFb, tumor necrosis factor beta; TRAIL, TNF-related apoptosis-inducing ligand;CRP,c-reactive protein;MIF,Macrophage migration inhibitory factor;CXCL9,chemokine (C-X-C motif) ligand 9.


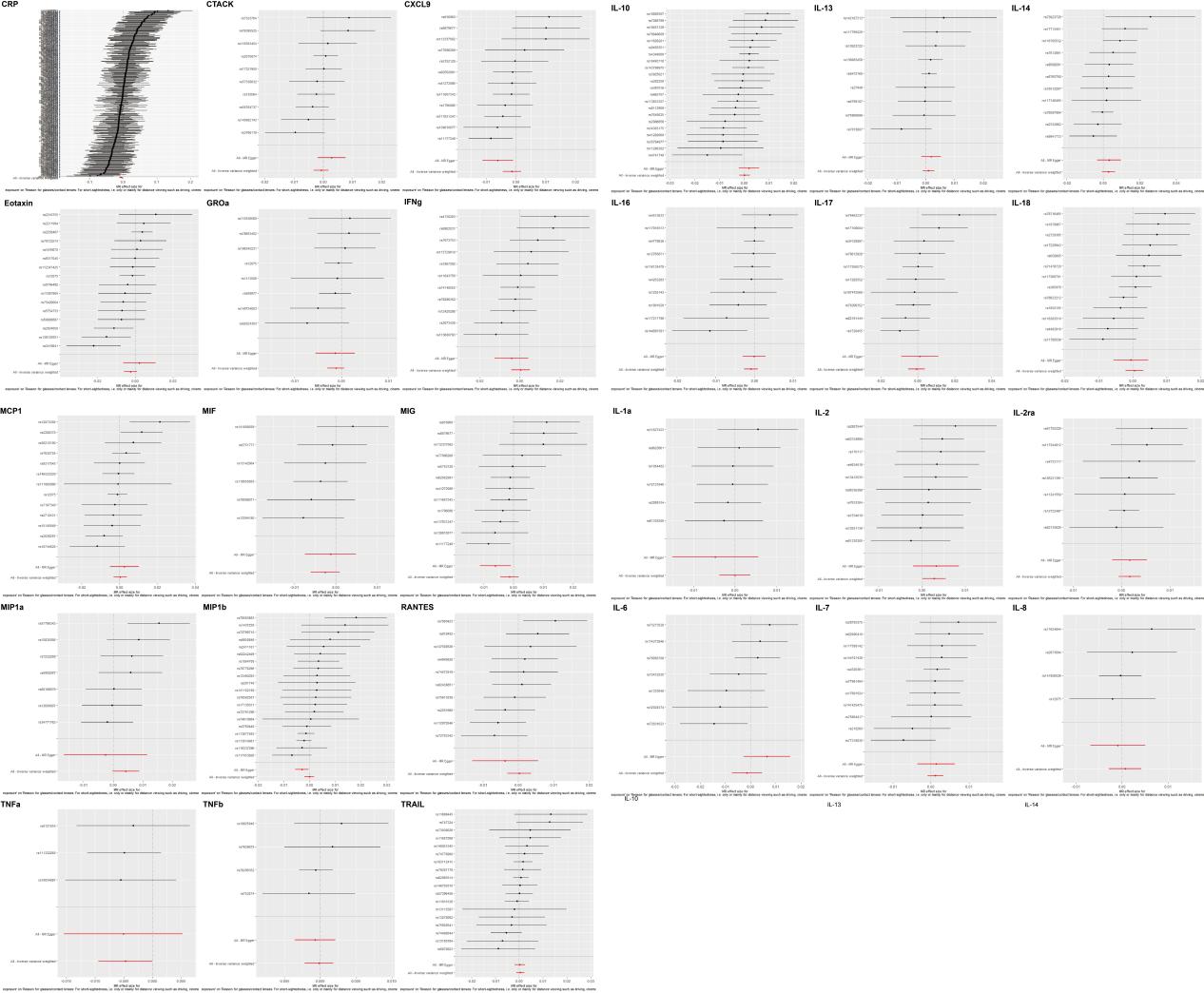


**S4. Forest plot of SNPs associated with inflammatory factors and their risk of myopia.**

Abbreviations:IVW, inversevariance weighted; MR, Mendelian randomization;

MR-PRESSO,MR Pleiotropy RESidual Sum and Outlier;SNP, single-nucleotide polymorphisms;CTACK, cutaneous T cell-attracting chemokine; GROa, growth-regulated oncogene-a; IFNg,interferon gamma;IL, interleukin;MCP1, monocyte chemotactic protein 1; MCP3, monocyte-specific chemokine 3; MIG, monokine induced by interferon gamma;MIP1a, macrophage inflammatory protein-1a; MIP1b, macrophage inflammatory protein-1b; TNFa, tumor necrosis factor alpha; TNFb, tumor necrosis factor beta; TRAIL, TNF-related apoptosis-inducing ligand;CRP,c-reactive protein;MIF,Macrophage migration inhibitory factor;CXCL9,chemokine (C-X-C motif) ligand 9.

**Table S5.All SNPs data in the manuscript.**

| **Inflammatory factors** | **No.** | **SNP** | **beta.exposure** | **beta.outcome** | **eaf.exposure** | **eaf.outcome** | **pval.outcome** | **se.exposure** | **pval.exposure** | **F** | **R2** |
| --- | --- | --- | --- | --- | --- | --- | --- | --- | --- | --- | --- |
| CRP | 1 | rs10169482 | -0.0143 | 0.000129471 | 0.399612 | 0.609029 | 0.82 | 0.0024 | 4.05E-09 | 35.50173611 | 9.81234E-05 |
| CRP | 2 | rs10408993 | 0.0332 | -0.000147866 | 0.055316621 | 0.944046 | 0.9 | 0.0051 | 4.95E-11 | 42.3775471 | 0.000115199 |
| CRP | 3 | rs10415983 | -0.024 | 0.00100087 | 0.162925049 | 0.836585 | 0.19 | 0.0032 | 3.82E-14 | 56.25 | 0.00015711 |
| CRP | 4 | rs10417602 | -0.0354 | 0.000824024 | 0.076251441 | 0.924085 | 0.44 | 0.0045 | 2.14E-15 | 61.88444444 | 0.000176538 |
| CRP | 5 | rs10493377 | -0.0209 | -0.000211412 | 0.45522931 | 0.545727 | 0.709999 | 0.0024 | 1.57E-18 | 75.83506944 | 0.000216654 |
| CRP | 6 | rs10501349 | -0.023 | -0.0016732 | 0.125954915 | 0.875337 | 0.051 | 0.0037 | 4.31E-10 | 38.64134405 | 0.000116476 |
| CRP | 7 | rs10521222 | -0.1014 | -0.000217994 | 0.047815444 | 0.951613 | 0.87 | 0.0056 | 9.19E-73 | 327.8686224 | 0.000936257 |
| CRP | 8 | rs1052373 | -0.024 | -0.00243459 | 0.322462752 | 0.678174 | 5.90E-05 | 0.0026 | 7.16E-21 | 85.20710059 | 0.00025169 |
| CRP | 9 | rs1064725 | 0.0472 | -0.00123549 | 0.041512472 | 0.958374 | 0.4 | 0.0062 | 4.08E-14 | 57.95629553 | 0.000177288 |
| CRP | 10 | rs10810455 | -0.0145 | 0.00152321 | 0.433762 | 0.440976 | 0.00769999 | 0.0024 | 1.90E-09 | 36.50173611 | 0.00010328 |
| CRP | 11 | rs10849772 | -0.0759 | -0.00087321 | 0.044945088 | 0.953812 | 0.52 | 0.0055 | 1.16E-43 | 190.44 | 0.000494566 |
| CRP | 12 | rs10851685 | 0.0219 | -0.000325654 | 0.133916308 | 0.135374 | 0.69 | 0.0035 | 3.05E-10 | 39.15183673 | 0.000111253 |
| CRP | 13 | rs10951261 | -0.0187 | -0.00142561 | 0.275415416 | 0.728455 | 0.025 | 0.0027 | 3.96E-12 | 47.96844993 | 0.00013957 |
| CRP | 14 | rs11039798 | -0.0284 | -0.00261372 | 0.129007194 | 0.872725 | 0.0021 | 0.0036 | 6.32E-15 | 62.2345679 | 0.000181257 |
| CRP | 15 | rs11065079 | -0.0876 | 0.000841496 | 0.020523562 | 0.979495 | 0.67 | 0.0085 | 9.59E-25 | 106.2112111 | 0.000308521 |
| CRP | 16 | rs11065219 | -0.0277 | -0.00171253 | 0.104504537 | 0.895662 | 0.064 | 0.0039 | 1.55E-12 | 50.44641683 | 0.000143611 |
| CRP | 17 | rs11065387 | -0.116 | 0.00180309 | 0.023751646 | 0.024147 | 0.33 | 0.0079 | 5.93E-49 | 215.6064733 | 0.000624022 |
| CRP | 18 | rs11077359 | 0.0307 | 3.79E-06 | 0.208813495 | 0.20917 | 1 | 0.0029 | 5.53E-26 | 112.0677765 | 0.000311418 |
| CRP | 19 | rs111231035 | 0.0829 | -0.00346828 | 0.024181426 | 0.975716 | 0.0599998 | 0.0078 | 3.34E-26 | 112.9587442 | 0.000324332 |
| CRP | 20 | rs11203042 | -0.0145 | -0.000338455 | 0.447186265 | 0.446984 | 0.55 | 0.0024 | 1.29E-09 | 36.50173611 | 0.000103952 |
| CRP | 21 | rs11208629 | -0.0323 | 0.000626712 | 0.080783679 | 0.919147 | 0.55 | 0.0044 | 2.40E-13 | 53.88894628 | 0.000154945 |
| CRP | 22 | rs11246574 | -0.0272 | -0.00193487 | 0.117167664 | 0.883983 | 0.0290001 | 0.0038 | 9.72E-13 | 51.23545706 | 0.000153057 |
| CRP | 23 | rs11249926 | 0.0151 | 0.00131254 | 0.489001561 | 0.49015 | 0.021 | 0.0024 | 3.32E-10 | 39.58506944 | 0.00011395 |
| CRP | 24 | rs112585178 | -0.0658 | 0.000908409 | 0.03237234 | 0.967776 | 0.57 | 0.0068 | 2.65E-22 | 93.63408304 | 0.000271247 |
| CRP | 25 | rs11265260 | 0.2137 | -0.00155504 | 0.057145209 | 0.942047 | 0.2 | 0.0051 | 1.00E-200 | 1755.774318 | 0.004921117 |
| CRP | 26 | rs11265608 | 0.0438 | 0.00021681 | 0.103417781 | 0.897453 | 0.82 | 0.0039 | 6.39E-29 | 126.1301775 | 0.000355765 |
| CRP | 27 | rs112749520 | -0.0853 | -0.00206611 | 0.015738535 | 0.984633 | 0.37 | 0.0098 | 2.75E-18 | 75.76103707 | 0.000225425 |
| CRP | 28 | rs113116967 | -0.0336 | -0.00150172 | 0.054529339 | 0.946827 | 0.23 | 0.0053 | 2.63E-10 | 40.19081524 | 0.000116409 |
| CRP | 29 | rs113188187 | 0.105 | -0.00127992 | 0.022636925 | 0.976805 | 0.5 | 0.008 | 2.94E-39 | 172.265625 | 0.000487845 |
| CRP | 30 | rs113401670 | -0.062 | 0.00230148 | 0.019112372 | 0.981417 | 0.27 | 0.0088 | 2.35E-12 | 49.63842975 | 0.000144128 |
| CRP | 31 | rs113405869 | 0.0218 | 0.000692805 | 0.126280178 | 0.873612 | 0.42 | 0.0036 | 1.82E-09 | 36.66975309 | 0.00010487 |
| CRP | 32 | rs113467192 | 0.0754 | -8.31E-05 | 0.021004176 | 0.978711 | 0.97 | 0.0083 | 7.02E-20 | 82.52518508 | 0.000233808 |
| CRP | 33 | rs113838402 | -0.0483 | 0.00204781 | 0.025923091 | 0.974526 | 0.25 | 0.0075 | 9.27E-11 | 41.4736 | 0.000117816 |
| CRP | 34 | rs114272969 | 0.0962 | -0.00433179 | 0.011648158 | 0.988513 | 0.1 | 0.0113 | 1.59E-17 | 72.47583992 | 0.000213083 |
| CRP | 35 | rs114530473 | 0.1034 | -0.00248805 | 0.023204184 | 0.976374 | 0.18 | 0.008 | 1.68E-38 | 167.055625 | 0.000484664 |
| CRP | 36 | rs114669363 | -0.0653 | 0.00258656 | 0.017724221 | 0.982648 | 0.23 | 0.0092 | 1.33E-12 | 50.37913516 | 0.000148476 |
| CRP | 37 | rs114695117 | -0.049 | -0.00271765 | 0.026641338 | 0.973546 | 0.12 | 0.0075 | 7.97E-11 | 42.68444444 | 0.000124523 |
| CRP | 38 | rs115165870 | 0.0431 | 0.00073523 | 0.041058624 | 0.95912 | 0.61 | 0.0061 | 1.49E-12 | 49.92233271 | 0.000146279 |
| CRP | 39 | rs115236337 | 0.0329 | 0.00159107 | 0.067627139 | 0.933461 | 0.16 | 0.0048 | 9.52E-12 | 46.97960069 | 0.0001365 |
| CRP | 40 | rs115368096 | -0.0466 | -0.00147869 | 0.023727777 | 0.975817 | 0.42 | 0.0078 | 2.03E-09 | 35.69296515 | 0.000100607 |
| CRP | 41 | rs115381557 | 0.2123 | 0.00252442 | 0.009674794 | 0.989646 | 0.37 | 0.0123 | 2.24E-66 | 297.913213 | 0.000863673 |
| CRP | 42 | rs115585839 | 0.1081 | 0.00057134 | 0.021857721 | 0.978759 | 0.77 | 0.0083 | 8.09E-39 | 169.6270867 | 0.000499676 |
| CRP | 43 | rs115615753 | 0.0611 | 0.000948579 | 0.038863558 | 0.960899 | 0.52 | 0.0063 | 2.18E-22 | 94.05920887 | 0.000278895 |
| CRP | 44 | rs11570051 | -0.0361 | -0.00479877 | 0.055500324 | 0.948723 | 0.000329997 | 0.0056 | 1.09E-10 | 41.55644133 | 0.000136629 |
| CRP | 45 | rs1160984 | 0.0722 | 0.000475312 | 0.058187034 | 0.941601 | 0.7 | 0.0054 | 4.52E-41 | 178.7668038 | 0.000571341 |
| CRP | 46 | rs11611673 | -0.0915 | 0.00388824 | 0.025068869 | 0.974521 | 0.0299999 | 0.0076 | 5.32E-33 | 144.9489266 | 0.000409243 |
| CRP | 47 | rs11640223 | 0.0235 | 9.79E-05 | 0.209370688 | 0.788456 | 0.89 | 0.0029 | 1.52E-15 | 65.66587396 | 0.000182833 |
| CRP | 48 | rs116509635 | 0.0686 | 0.00237376 | 0.018153693 | 0.981691 | 0.26 | 0.009 | 2.03E-14 | 58.0982716 | 0.000167759 |
| CRP | 49 | rs116643857 | 0.0583 | 0.00364275 | 0.023681397 | 0.976248 | 0.05 | 0.0079 | 1.92E-13 | 54.46066336 | 0.000157169 |
| CRP | 50 | rs11665829 | -0.0366 | 0.00106428 | 0.376599067 | 0.623876 | 0.0690001 | 0.0025 | 6.21E-50 | 214.3296 | 0.000628983 |
| CRP | 51 | rs11667234 | 0.034 | 0.00319513 | 0.0449433 | 0.953386 | 0.017 | 0.0057 | 2.83E-09 | 35.58017852 | 9.92389E-05 |
| CRP | 52 | rs11668327 | 0.0778 | 0.00146659 | 0.175577856 | 0.175779 | 0.0519996 | 0.0033 | 7.05E-122 | 555.8163453 | 0.0017523 |
| CRP | 53 | rs116699695 | -0.0599 | -0.0024739 | 0.02117999 | 0.978398 | 0.2 | 0.0084 | 7.82E-13 | 50.85048186 | 0.000148769 |
| CRP | 54 | rs11673516 | -0.0154 | -7.40E-05 | 0.432226393 | 0.572256 | 0.9 | 0.0024 | 1.78E-10 | 41.17361111 | 0.000116401 |
| CRP | 55 | rs116805289 | 0.0601 | 0.00235859 | 0.023092635 | 0.977257 | 0.21 | 0.0081 | 1.03E-13 | 55.05273586 | 0.000162969 |
| CRP | 56 | rs11682713 | -0.0322 | 0.00122403 | 0.105913277 | 0.894653 | 0.18 | 0.0039 | 2.02E-16 | 68.16831032 | 0.000196368 |
| CRP | 57 | rs116877397 | -0.0909 | 0.00190731 | 0.012059565 | 0.987969 | 0.46 | 0.0109 | 5.51E-17 | 69.54641865 | 0.000196888 |
| CRP | 58 | rs116881341 | -0.0743 | 0.00103069 | 0.018463807 | 0.981801 | 0.630001 | 0.009 | 1.65E-16 | 68.15419753 | 0.000200095 |
| CRP | 59 | rs1169282 | -0.1074 | -0.000358945 | 0.027675567 | 0.973216 | 0.84 | 0.0074 | 3.29E-47 | 210.6420745 | 0.000620792 |
| CRP | 60 | rs1169721 | -0.0226 | -0.000363884 | 0.208958524 | 0.791706 | 0.6 | 0.0029 | 9.70E-15 | 60.73246136 | 0.000168852 |
| CRP | 61 | rs117198034 | 0.074 | 0.00115895 | 0.022289958 | 0.978531 | 0.55 | 0.0083 | 3.13E-19 | 79.4890405 | 0.000238678 |
| CRP | 62 | rs117264457 | 0.0944 | 0.00107879 | 0.025055015 | 0.975679 | 0.56 | 0.0078 | 5.80E-34 | 146.4720579 | 0.00043536 |
| CRP | 63 | rs117310449 | -0.2624 | 0.00121366 | 0.012029524 | 0.988029 | 0.64 | 0.0117 | 3.81E-112 | 502.9860472 | 0.001636628 |
| CRP | 64 | rs117326714 | 0.0494 | -0.00485594 | 0.026542774 | 0.973655 | 0.00599998 | 0.0076 | 6.02E-11 | 42.25 | 0.000126109 |
| CRP | 65 | rs117368206 | -0.0606 | -0.000266305 | 0.019873039 | 0.979681 | 0.89 | 0.0086 | 2.36E-12 | 49.65332612 | 0.000143061 |
| CRP | 66 | rs117436012 | -0.0309 | 0.000404484 | 0.056116629 | 0.943151 | 0.74 | 0.0052 | 3.32E-09 | 35.31102071 | 0.000101148 |
| CRP | 67 | rs117453824 | -0.0613 | -0.00350244 | 0.014860446 | 0.985012 | 0.13 | 0.0099 | 5.72E-10 | 38.33986328 | 0.000110022 |
| CRP | 68 | rs117468623 | 0.0578 | 0.000145825 | 0.01732858 | 0.983148 | 0.95 | 0.0093 | 5.30E-10 | 38.62689328 | 0.000113778 |
| CRP | 69 | rs117477328 | -0.0735 | -0.00305189 | 0.012522497 | 0.986687 | 0.22 | 0.0108 | 9.45E-12 | 46.31558642 | 0.000133605 |
| CRP | 70 | rs11770879 | -0.0324 | 0.00135672 | 0.065707259 | 0.934485 | 0.24 | 0.0049 | 3.76E-11 | 43.72178259 | 0.000128889 |
| CRP | 71 | rs118106133 | -0.1095 | -0.00315488 | 0.023305821 | 0.976958 | 0.0940005 | 0.008 | 1.41E-42 | 187.3476563 | 0.00054586 |
| CRP | 72 | rs12042284 | 0.0305 | 0.000418868 | 0.088417377 | 0.911649 | 0.67 | 0.0042 | 5.65E-13 | 52.73526077 | 0.000149956 |
| CRP | 73 | rs12042349 | -0.0466 | -1.64E-05 | 0.096226163 | 0.902983 | 0.99 | 0.004 | 1.94E-31 | 135.7225 | 0.000377707 |
| CRP | 74 | rs12044132 | -0.0527 | -6.99E-05 | 0.160510735 | 0.839832 | 0.93 | 0.0033 | 8.90E-59 | 255.0312213 | 0.000748463 |
| CRP | 75 | rs12048215 | 0.0373 | -0.00122892 | 0.116356545 | 0.884017 | 0.16 | 0.0037 | 7.88E-24 | 101.6281958 | 0.000286098 |
| CRP | 76 | rs12313817 | -0.047 | -0.00149885 | 0.038416646 | 0.960495 | 0.3 | 0.0061 | 8.73E-15 | 59.36576189 | 0.000163204 |
| CRP | 77 | rs12328794 | 0.0168 | 0.000883058 | 0.488724634 | 0.510061 | 0.12 | 0.0024 | 1.92E-12 | 49 | 0.000141048 |
| CRP | 78 | rs12421530 | 0.021 | -0.000991736 | 0.38402725 | 0.385173 | 0.089 | 0.0024 | 8.40E-18 | 76.5625 | 0.000208637 |
| CRP | 79 | rs12601655 | 0.0288 | -8.71E-05 | 0.193763291 | 0.805088 | 0.9 | 0.003 | 1.01E-21 | 92.16 | 0.000259149 |
| CRP | 80 | rs12610605 | 0.0414 | -0.00130983 | 0.179574187 | 0.820603 | 0.0779992 | 0.0033 | 8.29E-37 | 157.3884298 | 0.000505026 |
| CRP | 81 | rs12625630 | 0.0185 | 0.000371535 | 0.174355804 | 0.825576 | 0.62 | 0.0032 | 4.29E-09 | 33.42285156 | 9.85378E-05 |
| CRP | 82 | rs12691088 | -0.2159 | -0.00354879 | 0.023730813 | 0.97911 | 0.0949992 | 0.0084 | 8.08E-145 | 660.6123866 | 0.00215982 |
| CRP | 83 | rs12721063 | 0.0682 | -0.00109195 | 0.011039021 | 0.98903 | 0.69 | 0.0116 | 3.64E-09 | 34.56629013 | 0.000101557 |
| CRP | 84 | rs12721109 | 0.0764 | 0.0010148 | 0.026031724 | 0.974436 | 0.57 | 0.0076 | 8.70E-24 | 101.0554017 | 0.000295981 |
| CRP | 85 | rs12728998 | -0.0197 | -0.00044461 | 0.172464835 | 0.825348 | 0.55 | 0.0031 | 3.84E-10 | 40.38397503 | 0.000110777 |
| CRP | 86 | rs12745083 | 0.1132 | 0.00186619 | 0.023184897 | 0.976813 | 0.32 | 0.0076 | 1.27E-50 | 221.8531856 | 0.000580417 |
| CRP | 87 | rs12816397 | 0.075 | -0.000143314 | 0.053093304 | 0.947658 | 0.91 | 0.0054 | 1.60E-43 | 192.9012346 | 0.000565587 |
| CRP | 88 | rs12819210 | 0.0596 | 0.000631943 | 0.18720866 | 0.813176 | 0.38 | 0.0031 | 4.79E-84 | 369.6316337 | 0.001081005 |
| CRP | 89 | rs12823525 | -0.0164 | 0.000560777 | 0.472957609 | 0.471891 | 0.32 | 0.0024 | 6.22E-12 | 46.69444444 | 0.000134087 |
| CRP | 90 | rs12941913 | 0.0155 | -0.000827359 | 0.399204 | 0.393431 | 0.15 | 0.0024 | 2.02E-10 | 41.71006944 | 0.000115243 |
| CRP | 91 | rs13016086 | -0.0302 | 0.00103087 | 0.119129384 | 0.882753 | 0.24 | 0.0038 | 1.00E-15 | 63.16066482 | 0.000191415 |
| CRP | 92 | rs13266821 | -0.0209 | 0.000300999 | 0.147763149 | 0.852526 | 0.709999 | 0.0034 | 4.42E-10 | 37.78633218 | 0.000110014 |
| CRP | 93 | rs13279515 | -0.0314 | -0.000467875 | 0.139982114 | 0.858538 | 0.57 | 0.0035 | 2.44E-19 | 80.48653061 | 0.000237394 |
| CRP | 94 | rs13374371 | 0.0259 | 0.00030881 | 0.122418673 | 0.87838 | 0.719999 | 0.0036 | 1.01E-12 | 51.76003086 | 0.000144133 |
| CRP | 95 | rs1371614 | -0.0227 | 0.000319853 | 0.245265177 | 0.754238 | 0.630001 | 0.0028 | 2.00E-16 | 65.72576531 | 0.000190771 |
| CRP | 96 | rs1386821 | -0.0619 | 0.000381546 | 0.204518165 | 0.796157 | 0.59 | 0.003 | 4.00E-97 | 425.7344444 | 0.001246733 |
| CRP | 97 | rs141622900 | 0.0921 | 0.00021287 | 0.052238408 | 0.947566 | 0.87 | 0.0056 | 8.62E-61 | 270.4850128 | 0.000839921 |
| CRP | 98 | rs141739979 | 0.0787 | -0.000326202 | 0.010639255 | 0.990494 | 0.92 | 0.0124 | 2.45E-10 | 40.28154266 | 0.00013039 |
| CRP | 99 | rs144261139 | -0.2545 | -0.000530465 | 0.012186018 | 0.98795 | 0.84 | 0.0114 | 9.51E-110 | 498.3860419 | 0.001559346 |
| CRP | 100 | rs147707133 | 0.0903 | 0.00159 | 0.017150515 | 0.016917 | 0.49 | 0.0098 | 3.57E-20 | 84.90306122 | 0.000274897 |
| CRP | 101 | rs150639620 | 0.0597 | -0.00203522 | 0.050161952 | 0.948391 | 0.11 | 0.0057 | 6.53E-26 | 109.6980609 | 0.000339627 |
| CRP | 102 | rs151291132 | 0.074 | -0.000955401 | 0.025991952 | 0.974516 | 0.6 | 0.008 | 1.60E-20 | 85.5625 | 0.000277265 |
| CRP | 103 | rs1524107 | -0.0528 | -0.00137276 | 0.043352572 | 0.954819 | 0.32 | 0.0058 | 6.80E-20 | 82.87277051 | 0.000231241 |
| CRP | 104 | rs164910 | -0.0284 | -0.00132281 | 0.085319954 | 0.91397 | 0.19 | 0.0042 | 1.52E-11 | 45.72335601 | 0.000125889 |
| CRP | 105 | rs1653607 | 0.0302 | 0.00100291 | 0.108220181 | 0.106403 | 0.28 | 0.0039 | 1.21E-14 | 59.96318212 | 0.000176039 |
| CRP | 106 | rs16835630 | -0.0759 | -1.26E-05 | 0.017787253 | 0.982425 | 1 | 0.0091 | 9.06E-17 | 69.56659824 | 0.000201293 |
| CRP | 107 | rs16950101 | -0.0442 | 0.000464211 | 0.056923795 | 0.944235 | 0.709999 | 0.0052 | 1.09E-17 | 72.25 | 0.000209756 |
| CRP | 108 | rs17097193 | 0.0468 | -0.00356485 | 0.031035254 | 0.968214 | 0.0269998 | 0.0067 | 2.48E-12 | 48.79126754 | 0.00013173 |
| CRP | 109 | rs17128076 | -0.046 | 0.00165574 | 0.038620884 | 0.962093 | 0.26 | 0.0062 | 9.28E-14 | 55.04682622 | 0.000157131 |
| CRP | 110 | rs17138478 | 0.0329 | -0.000334174 | 0.128551168 | 0.871153 | 0.69 | 0.0036 | 2.69E-20 | 83.51929012 | 0.000242516 |
| CRP | 111 | rs17216525 | 0.035 | -0.00237186 | 0.0813804 | 0.919596 | 0.0230001 | 0.0044 | 9.63E-16 | 63.27479339 | 0.000183156 |
| CRP | 112 | rs17408658 | -0.0308 | 0.000427353 | 0.320575515 | 0.679665 | 0.48 | 0.0026 | 3.48E-33 | 140.3313609 | 0.000413241 |
| CRP | 113 | rs17434647 | 0.0565 | -5.58E-05 | 0.123075987 | 0.877319 | 0.95 | 0.0037 | 9.92E-54 | 233.1811541 | 0.000689068 |
| CRP | 114 | rs17457976 | 0.0593 | 4.06E-05 | 0.15156629 | 0.848628 | 0.96 | 0.0034 | 1.99E-69 | 304.1946367 | 0.000904399 |
| CRP | 115 | rs17459069 | -0.117 | 0.00166349 | 0.03987762 | 0.960248 | 0.25 | 0.0062 | 1.57E-80 | 356.1134235 | 0.001048232 |
| CRP | 116 | rs17616063 | -0.1287 | 0.000146011 | 0.076133871 | 0.925549 | 0.89 | 0.0046 | 7.72E-175 | 782.7830813 | 0.002330097 |
| CRP | 117 | rs17658057 | 0.0381 | 0.00114005 | 0.044906313 | 0.956099 | 0.41 | 0.0059 | 7.75E-11 | 41.700948 | 0.000124518 |
| CRP | 118 | rs17796841 | -0.0345 | 0.0010364 | 0.06487806 | 0.936042 | 0.37 | 0.0049 | 2.20E-12 | 49.57309454 | 0.000144422 |
| CRP | 119 | rs17817449 | 0.021 | -0.000107421 | 0.393743945 | 0.604742 | 0.85 | 0.0024 | 7.34E-18 | 76.5625 | 0.000210542 |
| CRP | 120 | rs1855981 | -0.0159 | 0.00109802 | 0.27014881 | 0.270768 | 0.0850002 | 0.0027 | 2.10E-09 | 34.67901235 | 9.96923E-05 |
| CRP | 121 | rs187353341 | -0.0637 | 0.000983243 | 0.019259966 | 0.981032 | 0.64 | 0.0088 | 5.75E-13 | 52.3978564 | 0.000153292 |
| CRP | 122 | rs191977866 | 0.0725 | -1.57E-05 | 0.01926792 | 0.980993 | 0.99 | 0.0088 | 1.75E-16 | 67.87512913 | 0.000198651 |
| CRP | 123 | rs2049045 | -0.0209 | -0.000218435 | 0.187838332 | 0.186229 | 0.760001 | 0.0031 | 1.50E-11 | 45.45369407 | 0.000133275 |
| CRP | 124 | rs204906 | 0.0619 | 0.00330754 | 0.032799514 | 0.967099 | 0.0369999 | 0.0067 | 2.49E-20 | 85.35553575 | 0.000243106 |
| CRP | 125 | rs2110944 | 0.0147 | 0.000466136 | 0.481339 | 0.470101 | 0.41 | 0.0024 | 8.31E-10 | 37.515625 | 0.000107895 |
| CRP | 126 | rs2111125 | -0.0394 | 4.39E-05 | 0.350518899 | 0.353184 | 0.94 | 0.0025 | 1.47E-55 | 248.3776 | 0.000706806 |
| CRP | 127 | rs2207132 | 0.044 | -0.00110632 | 0.033101573 | 0.966637 | 0.48 | 0.0067 | 6.63E-11 | 43.12764536 | 0.000123927 |
| CRP | 128 | rs2212339 | -0.0248 | -0.00180772 | 0.12964955 | 0.871167 | 0.032 | 0.0036 | 1.05E-11 | 47.45679012 | 0.000138803 |
| CRP | 129 | rs2235780 | -0.0715 | 0.00195779 | 0.030389096 | 0.030428 | 0.24 | 0.007 | 2.62E-24 | 104.3316327 | 0.000301271 |
| CRP | 130 | rs2244608 | -0.1388 | 0.00113281 | 0.315270643 | 0.681064 | 0.0619998 | 0.0026 | 1.00E-200 | 2849.91716 | 0.008317856 |
| CRP | 131 | rs2245407 | -0.0602 | 0.00052773 | 0.070775137 | 0.929583 | 0.630001 | 0.0045 | 3.69E-40 | 178.9649383 | 0.000476677 |
| CRP | 132 | rs2269434 | -0.0243 | -0.00202107 | 0.346475926 | 0.654594 | 0.000680002 | 0.0025 | 2.86E-22 | 94.4784 | 0.00026741 |
| CRP | 133 | rs2269841 | 0.0233 | 0.0009423 | 0.281872975 | 0.718624 | 0.14 | 0.0026 | 9.55E-19 | 80.3091716 | 0.000219784 |
| CRP | 134 | rs2280405 | -0.0154 | 0.00106916 | 0.465627246 | 0.46646 | 0.0589997 | 0.0024 | 9.50E-11 | 41.17361111 | 0.00011802 |
| CRP | 135 | rs2526353 | -0.021 | -0.00034881 | 0.198501228 | 0.803642 | 0.62 | 0.003 | 1.97E-12 | 49 | 0.000140325 |
| CRP | 136 | rs2686340 | 0.0257 | 0.00041723 | 0.197958098 | 0.198219 | 0.56 | 0.003 | 6.25E-18 | 73.38777778 | 0.000209733 |
| CRP | 137 | rs2722733 | 0.0176 | -0.000221323 | 0.237290641 | 0.236512 | 0.74 | 0.0028 | 5.44E-10 | 39.51020408 | 0.000112123 |
| CRP | 138 | rs2836878 | -0.0324 | -0.00125032 | 0.267162371 | 0.733629 | 0.051 | 0.0027 | 5.43E-33 | 144 | 0.000411058 |
| CRP | 139 | rs283814 | -0.0481 | 0.00101147 | 0.07802237 | 0.077946 | 0.34 | 0.0045 | 7.94E-27 | 114.2523457 | 0.000332859 |
| CRP | 140 | rs28399637 | -0.059 | -0.000782018 | 0.317274968 | 0.684715 | 0.2 | 0.0026 | 4.21E-116 | 514.9408284 | 0.00150805 |
| CRP | 141 | rs28489942 | -0.0177 | 0.000807972 | 0.350315726 | 0.651172 | 0.17 | 0.0025 | 1.73E-12 | 50.1264 | 0.000142606 |
| CRP | 142 | rs2859400 | -0.0259 | 0.000367271 | 0.163574142 | 0.16414 | 0.630001 | 0.0032 | 5.18E-16 | 65.50878906 | 0.000183557 |
| CRP | 143 | rs2927437 | 0.0345 | 0.000949204 | 0.19274926 | 0.191429 | 0.19 | 0.003 | 5.25E-30 | 132.25 | 0.000370399 |
| CRP | 144 | rs2965164 | 0.0245 | 9.00E-05 | 0.345785537 | 0.653919 | 0.88 | 0.0025 | 1.72E-22 | 96.04 | 0.000271575 |
| CRP | 145 | rs3027067 | -0.0892 | 0.00111102 | 0.041187179 | 0.958408 | 0.43 | 0.006 | 2.05E-49 | 221.0177778 | 0.000628428 |
| CRP | 146 | rs3093070 | 0.1019 | 0.00481056 | 0.017918778 | 0.982359 | 0.025 | 0.0091 | 4.83E-29 | 125.3907741 | 0.000365455 |
| CRP | 147 | rs315946 | -0.0253 | -0.000141982 | 0.144713778 | 0.855856 | 0.86 | 0.0034 | 1.75E-13 | 55.37110727 | 0.00015845 |
| CRP | 148 | rs34095326 | -0.1919 | 0.0016951 | 0.113927004 | 0.886861 | 0.0589997 | 0.004 | 1.00E-200 | 2301.600625 | 0.007434917 |
| CRP | 149 | rs34179846 | 0.0472 | 0.000553016 | 0.104540514 | 0.895375 | 0.55 | 0.0039 | 3.52E-33 | 146.4720579 | 0.000417104 |
| CRP | 150 | rs34473506 | 0.0268 | -0.00112966 | 0.156676324 | 0.841884 | 0.15 | 0.0033 | 3.15E-16 | 65.95408632 | 0.0001898 |
| CRP | 151 | rs34693607 | -0.0334 | -0.00125292 | 0.213577778 | 0.212409 | 0.0700003 | 0.0029 | 5.27E-30 | 132.646849 | 0.000374744 |
| CRP | 152 | rs34982954 | -0.0147 | -0.000189011 | 0.474337842 | 0.527696 | 0.74 | 0.0024 | 9.07E-10 | 37.515625 | 0.00010776 |
| CRP | 153 | rs35315097 | 0.0508 | 0.000427078 | 0.053496183 | 0.946107 | 0.73 | 0.0054 | 2.26E-21 | 88.49931413 | 0.000261338 |
| CRP | 154 | rs35860194 | 0.0635 | -0.00075248 | 0.015833499 | 0.984105 | 0.74 | 0.0096 | 4.12E-11 | 43.75271267 | 0.000125667 |
| CRP | 155 | rs36227 | 0.02 | -0.000891667 | 0.180224443 | 0.181039 | 0.23 | 0.0031 | 1.14E-10 | 41.62330905 | 0.000118195 |
| CRP | 156 | rs365653 | 0.0644 | -0.00108016 | 0.114569393 | 0.884272 | 0.23 | 0.0039 | 6.49E-62 | 272.6732413 | 0.000841443 |
| CRP | 157 | rs3745150 | 0.038 | -0.000831219 | 0.39392291 | 0.393633 | 0.16 | 0.0026 | 3.35E-49 | 213.6094675 | 0.000689503 |
| CRP | 158 | rs3781619 | -0.0315 | -0.00193564 | 0.155155667 | 0.84523 | 0.0129999 | 0.0033 | 6.07E-22 | 91.11570248 | 0.000260133 |
| CRP | 159 | rs3812814 | 0.0247 | 0.000766978 | 0.122842151 | 0.878629 | 0.38 | 0.0037 | 1.27E-11 | 44.56464573 | 0.000131477 |
| CRP | 160 | rs3815989 | -0.0267 | 0.000819365 | 0.11339958 | 0.88551 | 0.36 | 0.0037 | 8.56E-13 | 52.07377648 | 0.000143348 |
| CRP | 161 | rs3850515 | -0.0654 | -0.000380095 | 0.013760228 | 0.014127 | 0.87 | 0.0093 | 2.08E-12 | 49.45265349 | 0.00011609 |
| CRP | 162 | rs405697 | -0.062 | 0.00150177 | 0.276026832 | 0.27594 | 0.0189998 | 0.0028 | 1.16E-107 | 490.3061224 | 0.001536339 |
| CRP | 163 | rs41264481 | 0.1018 | 0.000836129 | 0.017669403 | 0.982662 | 0.7 | 0.009 | 2.29E-29 | 127.9412346 | 0.000359754 |
| CRP | 164 | rs41289512 | -0.1788 | 0.000779675 | 0.043305802 | 0.042348 | 0.58 | 0.0059 | 1.25E-198 | 918.3981614 | 0.002649014 |
| CRP | 165 | rs41290100 | 0.071 | -0.000411806 | 0.029858706 | 0.971851 | 0.82 | 0.0075 | 2.02E-21 | 89.61777778 | 0.000292047 |
| CRP | 166 | rs41310887 | 0.0404 | -0.00209673 | 0.048358189 | 0.951701 | 0.11 | 0.0056 | 6.22E-13 | 52.04591837 | 0.000150223 |
| CRP | 167 | rs41310893 | 0.045 | -0.000900301 | 0.049845481 | 0.950975 | 0.49 | 0.0056 | 5.78E-16 | 64.57270408 | 0.000191812 |
| CRP | 168 | rs4131568 | 0.0915 | -0.0011882 | 0.338026178 | 0.662045 | 0.0479999 | 0.0025 | 1.00E-200 | 1339.56 | 0.003746824 |
| CRP | 169 | rs416041 | 0.0361 | -0.000915709 | 0.375985784 | 0.374966 | 0.12 | 0.0026 | 7.91E-44 | 192.7825444 | 0.000611519 |
| CRP | 170 | rs4282816 | -0.0175 | -0.000162612 | 0.261392916 | 0.262454 | 0.8 | 0.0027 | 7.55E-11 | 42.00960219 | 0.000118253 |
| CRP | 171 | rs4394621 | 0.1088 | -0.000174333 | 0.184154093 | 0.18231 | 0.81 | 0.0031 | 1.00E-200 | 1231.783559 | 0.003556946 |
| CRP | 172 | rs4411129 | 0.0178 | 0.0001015 | 0.292615757 | 0.292978 | 0.87 | 0.0026 | 1.38E-11 | 46.86982249 | 0.000131167 |
| CRP | 173 | rs4420638 | -0.2217 | 0.00224756 | 0.191027615 | 0.811139 | 0.00189998 | 0.003 | 1.00E-200 | 5461.21 | 0.01519117 |
| CRP | 174 | rs4527079 | -0.0268 | 7.86E-05 | 0.078802039 | 0.079553 | 0.94 | 0.0044 | 1.35E-09 | 37.09917355 | 0.000104277 |
| CRP | 175 | rs45446698 | -0.0425 | -0.00122761 | 0.042780939 | 0.958111 | 0.39 | 0.006 | 1.11E-12 | 50.17361111 | 0.000147935 |
| CRP | 176 | rs4714508 | 0.0198 | -0.000650622 | 0.351713 | 0.653733 | 0.28 | 0.0025 | 5.40E-15 | 62.7264 | 0.000178779 |
| CRP | 177 | rs4767878 | 0.0518 | -0.00207518 | 0.026684643 | 0.026301 | 0.24 | 0.0075 | 4.90E-12 | 47.70204444 | 0.000139381 |
| CRP | 178 | rs4767915 | -0.0347 | 0.000814243 | 0.097756958 | 0.902207 | 0.39 | 0.004 | 5.72E-18 | 75.255625 | 0.000212403 |
| CRP | 179 | rs4806073 | -0.0286 | -0.00204469 | 0.066765561 | 0.067295 | 0.0700003 | 0.0045 | 3.27E-10 | 40.39308642 | 0.000101931 |
| CRP | 180 | rs483082 | -0.152 | 0.00146436 | 0.237110595 | 0.76476 | 0.0290001 | 0.0029 | 1.00E-200 | 2747.205707 | 0.008358526 |
| CRP | 181 | rs4916009 | 0.0335 | 0.00219218 | 0.091693935 | 0.909817 | 0.0259998 | 0.0042 | 1.37E-15 | 63.61961451 | 0.000186936 |
| CRP | 182 | rs4925671 | 0.0194 | -0.000546334 | 0.302022351 | 0.302582 | 0.38 | 0.0026 | 6.44E-14 | 55.67455621 | 0.000158677 |
| CRP | 183 | rs507666 | 0.0329 | 0.000352206 | 0.184602484 | 0.815291 | 0.630001 | 0.0031 | 2.95E-26 | 112.6337149 | 0.000325858 |
| CRP | 184 | rs55688443 | -0.0973 | -0.000596671 | 0.021692872 | 0.977788 | 0.760001 | 0.0081 | 2.67E-33 | 144.2964487 | 0.000401835 |
| CRP | 185 | rs55997241 | 0.0315 | -0.000505285 | 0.076439214 | 0.923409 | 0.64 | 0.0046 | 5.18E-12 | 46.89272212 | 0.000140098 |
| CRP | 186 | rs56156770 | 0.0845 | 0.00430402 | 0.021813905 | 0.978359 | 0.0269998 | 0.0083 | 1.90E-24 | 103.6471186 | 0.000304718 |
| CRP | 187 | rs56401401 | -0.0729 | -0.00246493 | 0.032838882 | 0.966934 | 0.12 | 0.0067 | 1.54E-27 | 118.3873914 | 0.000337577 |
| CRP | 188 | rs572144 | -0.014 | 9.50E-05 | 0.459977524 | 0.539944 | 0.87 | 0.0024 | 4.66E-09 | 34.02777778 | 9.73721E-05 |
| CRP | 189 | rs584007 | -0.0901 | 0.00181889 | 0.355942076 | 0.35974 | 0.0021 | 0.0026 | 1.00E-200 | 1200.889053 | 0.003722064 |
| CRP | 190 | rs59104589 | 0.0183 | -3.80E-05 | 0.355894 | 0.641349 | 0.95 | 0.0025 | 1.49E-13 | 53.5824 | 0.000153536 |
| CRP | 191 | rs60049679 | -0.1277 | -0.0027276 | 0.07720473 | 0.073126 | 0.0189998 | 0.0046 | 3.68E-169 | 770.665879 | 0.002323598 |
| CRP | 192 | rs60517797 | 0.0325 | -0.0017445 | 0.08025554 | 0.918538 | 0.0920005 | 0.0043 | 6.66E-14 | 57.12547323 | 0.000155933 |
| CRP | 193 | rs61753391 | 0.0515 | -0.0027692 | 0.026332124 | 0.974011 | 0.12 | 0.0076 | 9.44E-12 | 45.91845568 | 0.000136001 |
| CRP | 194 | rs61806853 | -0.0452 | 0.000607158 | 0.049732908 | 0.950555 | 0.64 | 0.0055 | 3.80E-16 | 67.5385124 | 0.000193106 |
| CRP | 195 | rs61811421 | 0.0396 | -0.000551699 | 0.22577334 | 0.773703 | 0.42 | 0.0029 | 1.17E-43 | 186.4637337 | 0.000548228 |
| CRP | 196 | rs61821592 | -0.08 | 0.000185079 | 0.021472593 | 0.97916 | 0.93 | 0.0084 | 1.51E-21 | 90.70294785 | 0.000268947 |
| CRP | 197 | rs62023507 | -0.0329 | -0.000674706 | 0.065627089 | 0.934297 | 0.55 | 0.0048 | 9.16E-12 | 46.97960069 | 0.000132747 |
| CRP | 198 | rs62117160 | 0.0587 | -2.46E-05 | 0.04595752 | 0.954756 | 0.99 | 0.0058 | 3.48E-24 | 102.4283591 | 0.000302155 |
| CRP | 199 | rs62513191 | 0.036 | -0.00129116 | 0.043681928 | 0.957071 | 0.35 | 0.0059 | 1.21E-09 | 37.23068084 | 0.000108278 |
| CRP | 200 | rs6501734 | -0.028 | 0.0010761 | 0.105524826 | 0.895001 | 0.24 | 0.0038 | 2.69E-13 | 54.29362881 | 0.000148002 |
| CRP | 201 | rs6573778 | 0.0153 | 0.000228446 | 0.472722 | 0.481156 | 0.69 | 0.0024 | 1.78E-10 | 40.640625 | 0.000116697 |
| CRP | 202 | rs6672331 | 0.1248 | -0.00243887 | 0.032780996 | 0.032965 | 0.12 | 0.0068 | 4.75E-76 | 336.8304498 | 0.000987657 |
| CRP | 203 | rs6698040 | -0.0454 | 0.000423472 | 0.223811265 | 0.225559 | 0.53 | 0.0028 | 8.50E-58 | 262.9030612 | 0.000716129 |
| CRP | 204 | rs672140 | -0.0394 | -0.000349104 | 0.078364989 | 0.921849 | 0.74 | 0.0043 | 1.02E-19 | 83.95673337 | 0.000224235 |
| CRP | 205 | rs6728590 | 0.0348 | -0.000966506 | 0.485252066 | 0.515167 | 0.0879995 | 0.0025 | 1.51E-43 | 193.7664 | 0.000604993 |
| CRP | 206 | rs6792725 | -0.0195 | -0.000316747 | 0.30437 | 0.307611 | 0.62 | 0.0027 | 5.12E-13 | 52.16049383 | 0.00016102 |
| CRP | 207 | rs6859 | 0.0548 | -0.000800835 | 0.42245825 | 0.421562 | 0.16 | 0.0024 | 6.46E-115 | 521.3611111 | 0.001465407 |
| CRP | 208 | rs6986685 | 0.0198 | 0.00103512 | 0.179274583 | 0.823505 | 0.16 | 0.0032 | 4.28E-10 | 38.28515625 | 0.000115366 |
| CRP | 209 | rs7084062 | 0.017 | 0.00143686 | 0.491779 | 0.507794 | 0.012 | 0.0024 | 1.49E-12 | 50.17361111 | 0.000144461 |
| CRP | 210 | rs71441083 | 0.0556 | -0.00117553 | 0.033732261 | 0.965834 | 0.450001 | 0.0066 | 6.02E-17 | 70.96786042 | 0.000201522 |
| CRP | 211 | rs71454665 | 0.0718 | 0.000309813 | 0.057363828 | 0.94278 | 0.8 | 0.0052 | 5.19E-44 | 190.6523669 | 0.000557521 |
| CRP | 212 | rs714948 | -0.0405 | 0.000339347 | 0.117267995 | 0.884285 | 0.7 | 0.0038 | 4.88E-27 | 113.5907202 | 0.000339585 |
| CRP | 213 | rs71556711 | -0.0292 | 0.000983991 | 0.090616444 | 0.910163 | 0.32 | 0.0042 | 3.39E-12 | 48.33560091 | 0.000140524 |
| CRP | 214 | rs7223548 | 0.0282 | 0.000196767 | 0.101461126 | 0.103105 | 0.83 | 0.0039 | 3.18E-13 | 52.28402367 | 0.000144999 |
| CRP | 215 | rs7254892 | 0.0884 | 0.00101683 | 0.031391532 | 0.968308 | 0.53 | 0.0066 | 1.64E-40 | 179.3976125 | 0.000475221 |
| CRP | 216 | rs72654472 | 0.0737 | -0.00429094 | 0.026787357 | 0.973041 | 0.0140001 | 0.0075 | 9.35E-23 | 96.56337778 | 0.000283206 |
| CRP | 217 | rs72667426 | -0.0488 | 0.000151494 | 0.025804852 | 0.974686 | 0.93 | 0.0077 | 1.90E-10 | 40.16596391 | 0.000119734 |
| CRP | 218 | rs72679119 | -0.0357 | 0.000705683 | 0.133232921 | 0.13213 | 0.4 | 0.0036 | 1.06E-23 | 98.34027778 | 0.000294361 |
| CRP | 219 | rs72685056 | -0.0506 | -0.000966655 | 0.128436018 | 0.873005 | 0.26 | 0.0036 | 2.76E-44 | 197.558642 | 0.000573214 |
| CRP | 220 | rs72698555 | -0.0538 | 0.000675199 | 0.079713651 | 0.919968 | 0.52 | 0.0044 | 2.44E-34 | 149.5061983 | 0.000424669 |
| CRP | 221 | rs72698573 | -0.09 | 0.000734658 | 0.085691292 | 0.914603 | 0.47 | 0.0043 | 3.55E-97 | 438.0746349 | 0.001269242 |
| CRP | 222 | rs72743115 | -0.0479 | 0.000491827 | 0.041409958 | 0.958944 | 0.73 | 0.0061 | 2.88E-15 | 61.6611126 | 0.000182154 |
| CRP | 223 | rs72814468 | 0.0277 | -0.000664652 | 0.126974956 | 0.874854 | 0.44 | 0.0036 | 2.32E-14 | 59.20447531 | 0.000170112 |
| CRP | 224 | rs72961013 | -0.0495 | 0.00142014 | 0.067125985 | 0.934425 | 0.21 | 0.0049 | 2.40E-24 | 102.0512287 | 0.00030687 |
| CRP | 225 | rs73020704 | -0.0582 | 0.00204194 | 0.019583608 | 0.980249 | 0.32 | 0.0082 | 1.41E-12 | 50.3753718 | 0.000130071 |
| CRP | 226 | rs7302482 | 0.0647 | -0.000363662 | 0.076096022 | 0.923566 | 0.73 | 0.0044 | 1.99E-48 | 216.223657 | 0.00058861 |
| CRP | 227 | rs73050293 | 0.0223 | 0.00122503 | 0.131601664 | 0.869311 | 0.15 | 0.0038 | 3.33E-09 | 34.43836565 | 0.000113663 |
| CRP | 228 | rs7305618 | 0.0886 | 3.79E-05 | 0.22819264 | 0.76993 | 0.96 | 0.0028 | 1.00E-200 | 1001.270408 | 0.002765082 |
| CRP | 229 | rs73157142 | -0.036 | 0.00054868 | 0.048799273 | 0.951145 | 0.68 | 0.0056 | 1.51E-10 | 41.32653061 | 0.000120315 |
| CRP | 230 | rs73214144 | 0.0364 | -0.00166969 | 0.133412172 | 0.868563 | 0.0460002 | 0.0035 | 7.96E-25 | 108.16 | 0.000306366 |
| CRP | 231 | rs73220272 | -0.0267 | 0.00093732 | 0.087054003 | 0.913281 | 0.35 | 0.0043 | 3.70E-10 | 38.55543537 | 0.000113315 |
| CRP | 232 | rs73921514 | -0.0369 | 0.00157743 | 0.038695222 | 0.961194 | 0.28 | 0.0061 | 1.21E-09 | 36.59258264 | 0.000101298 |
| CRP | 233 | rs74541405 | 0.0305 | -0.0010138 | 0.05574267 | 0.944083 | 0.41 | 0.0052 | 4.11E-09 | 34.40273669 | 9.79282E-05 |
| CRP | 234 | rs74580701 | -0.0383 | 0.000637449 | 0.04289069 | 0.956889 | 0.649999 | 0.0059 | 1.16E-10 | 42.13990233 | 0.000120435 |
| CRP | 235 | rs74587498 | -0.0356 | 0.000575199 | 0.052617916 | 0.947371 | 0.649999 | 0.0054 | 4.02E-11 | 43.46227709 | 0.000126354 |
| CRP | 236 | rs74607435 | 0.0447 | 0.000171334 | 0.051531678 | 0.949086 | 0.89 | 0.0055 | 3.42E-16 | 66.05256198 | 0.000195318 |
| CRP | 237 | rs74663951 | 0.0507 | -0.000196361 | 0.024805932 | 0.975324 | 0.91 | 0.0078 | 6.19E-11 | 42.25 | 0.000124363 |
| CRP | 238 | rs74876709 | 0.0795 | -0.000575799 | 0.018297218 | 0.981542 | 0.780001 | 0.009 | 1.13E-18 | 78.02777778 | 0.000227054 |
| CRP | 239 | rs75089355 | 0.0309 | -5.74E-06 | 0.145831167 | 0.854968 | 0.99 | 0.0034 | 7.15E-20 | 82.59602076 | 0.000237871 |
| CRP | 240 | rs75120785 | 0.0294 | 1.85E-05 | 0.101517672 | 0.898334 | 0.98 | 0.004 | 1.54E-13 | 54.0225 | 0.00015768 |
| CRP | 241 | rs75148473 | -0.0438 | 0.000546684 | 0.033310727 | 0.967388 | 0.73 | 0.0068 | 9.39E-11 | 41.48875433 | 0.000123552 |
| CRP | 242 | rs75164063 | -0.0512 | -0.00129922 | 0.024239874 | 0.975443 | 0.48 | 0.0078 | 6.90E-11 | 43.08744247 | 0.000124006 |
| CRP | 243 | rs7546735 | 0.0165 | 0.000717229 | 0.274638164 | 0.724053 | 0.26 | 0.0027 | 1.19E-09 | 37.34567901 | 0.000108471 |
| CRP | 244 | rs75564621 | -0.0274 | -0.000238023 | 0.082354898 | 0.91803 | 0.82 | 0.0044 | 3.60E-10 | 38.77892562 | 0.000113474 |
| CRP | 245 | rs7563362 | 0.0208 | 0.000847204 | 0.15202 | 0.14385 | 0.3 | 0.0034 | 8.23E-10 | 37.42560554 | 0.000111543 |
| CRP | 246 | rs7573232 | 0.0202 | -7.71E-05 | 0.326391925 | 0.674094 | 0.9 | 0.0026 | 2.67E-15 | 60.36094675 | 0.000179424 |
| CRP | 247 | rs75880130 | -0.0264 | -0.00244062 | 0.082400592 | 0.916612 | 0.017 | 0.0043 | 6.98E-10 | 37.69388859 | 0.000105395 |
| CRP | 248 | rs75976622 | -0.0386 | 0.00194018 | 0.0407901 | 0.039161 | 0.19 | 0.0061 | 2.52E-10 | 40.04192421 | 0.000116593 |
| CRP | 249 | rs7602171 | -0.0155 | -0.000530151 | 0.328445913 | 0.326919 | 0.38 | 0.0025 | 1.10E-09 | 38.44 | 0.000105983 |
| CRP | 250 | rs76164683 | 0.0592 | 0.00370598 | 0.018856074 | 0.018637 | 0.0769999 | 0.0089 | 3.06E-11 | 44.24491857 | 0.000129675 |
| CRP | 251 | rs76263497 | 0.0238 | 0.000956874 | 0.157955604 | 0.845117 | 0.22 | 0.0033 | 8.90E-13 | 52.01469238 | 0.000150679 |
| CRP | 252 | rs76365233 | 0.0247 | 3.60E-05 | 0.090740113 | 0.090388 | 0.97 | 0.0042 | 4.28E-09 | 34.58560091 | 0.000100673 |
| CRP | 253 | rs76366838 | -0.2294 | 0.000577496 | 0.026210309 | 0.973927 | 0.75 | 0.0078 | 2.17E-191 | 864.9631821 | 0.002686298 |
| CRP | 254 | rs76419583 | -0.0229 | -0.000926538 | 0.154182215 | 0.846815 | 0.24 | 0.0033 | 4.66E-12 | 48.15518825 | 0.000136777 |
| CRP | 255 | rs76475417 | -0.05 | 0.00169242 | 0.028800541 | 0.970824 | 0.31 | 0.0071 | 1.50E-12 | 49.59333466 | 0.000139855 |
| CRP | 256 | rs76476582 | 0.0347 | 0.00108417 | 0.055557205 | 0.944325 | 0.38 | 0.0053 | 5.51E-11 | 42.86543254 | 0.000126359 |
| CRP | 257 | rs76769546 | -0.0342 | -0.00187481 | 0.048256831 | 0.951976 | 0.16 | 0.0056 | 1.19E-09 | 37.29719388 | 0.000107439 |
| CRP | 258 | rs76841017 | -0.0561 | 0.000639725 | 0.028522511 | 0.971849 | 0.709999 | 0.0073 | 1.12E-14 | 59.05817226 | 0.000174412 |
| CRP | 259 | rs76856627 | -0.0567 | 0.00284195 | 0.033602586 | 0.967137 | 0.0739997 | 0.0068 | 5.07E-17 | 69.52616782 | 0.000208797 |
| CRP | 260 | rs76870318 | 0.056 | -0.00283006 | 0.020628264 | 0.979754 | 0.16 | 0.0085 | 5.49E-11 | 43.40484429 | 0.000126712 |
| CRP | 261 | rs76898938 | 0.0447 | 0.000536282 | 0.02608937 | 0.974376 | 0.760001 | 0.0076 | 3.78E-09 | 34.59297091 | 0.000101538 |
| CRP | 262 | rs76912411 | -0.0655 | -0.000257529 | 0.079515009 | 0.078893 | 0.81 | 0.0045 | 1.58E-48 | 211.8641975 | 0.000628027 |
| CRP | 263 | rs769449 | -0.2593 | 0.00166258 | 0.126903251 | 0.874951 | 0.0519996 | 0.0036 | 1.00E-200 | 5188.000772 | 0.014899447 |
| CRP | 264 | rs769450 | 0.0449 | 0.000525779 | 0.39615452 | 0.603573 | 0.36 | 0.0024 | 4.33E-76 | 350.0017361 | 0.000964524 |
| CRP | 265 | rs76962533 | -0.067 | -0.00115704 | 0.037851964 | 0.962034 | 0.43 | 0.0063 | 2.60E-26 | 113.1015369 | 0.000326972 |
| CRP | 266 | rs77273543 | 0.0497 | 0.00121921 | 0.05010843 | 0.050505 | 0.35 | 0.0055 | 2.79E-19 | 81.65586777 | 0.000235141 |
| CRP | 267 | rs77383163 | 0.0375 | -0.000648949 | 0.084650964 | 0.915605 | 0.52 | 0.0043 | 4.92E-18 | 76.05462412 | 0.000217927 |
| CRP | 268 | rs77451629 | 0.0445 | -0.000326076 | 0.028935364 | 0.971038 | 0.85 | 0.0072 | 7.04E-10 | 38.19926698 | 0.000111283 |
| CRP | 269 | rs77509028 | 0.0615 | -0.00111513 | 0.015364326 | 0.983945 | 0.62 | 0.0097 | 2.67E-10 | 40.19821448 | 0.000114438 |
| CRP | 270 | rs77532628 | -0.0366 | 0.000634412 | 0.051094369 | 0.948937 | 0.62 | 0.0054 | 1.55E-11 | 45.9382716 | 0.000129894 |
| CRP | 271 | rs77617917 | -0.0284 | -0.000720875 | 0.079561641 | 0.921107 | 0.49 | 0.0045 | 1.80E-10 | 39.83012346 | 0.000118131 |
| CRP | 272 | rs77704739 | -0.0532 | -0.00147838 | 0.041979102 | 0.958124 | 0.3 | 0.006 | 7.27E-19 | 78.61777778 | 0.000227647 |
| CRP | 273 | rs77801962 | -0.0643 | -0.00106709 | 0.029667388 | 0.970265 | 0.52 | 0.0071 | 1.96E-19 | 82.01725848 | 0.000238041 |
| CRP | 274 | rs77828979 | -0.0252 | -0.00172181 | 0.133298607 | 0.868208 | 0.04 | 0.0036 | 2.40E-12 | 49 | 0.000146732 |
| CRP | 275 | rs77993403 | -0.0415 | -0.000164881 | 0.048200386 | 0.952898 | 0.9 | 0.0057 | 2.16E-13 | 53.00861804 | 0.000158024 |
| CRP | 276 | rs77994054 | -0.0522 | 0.00198079 | 0.01924427 | 0.981174 | 0.34 | 0.0088 | 3.51E-09 | 35.18646694 | 0.000102857 |
| CRP | 277 | rs77994623 | 0.0476 | 0.000482797 | 0.166217429 | 0.832801 | 0.52 | 0.0032 | 2.90E-50 | 221.265625 | 0.00062802 |
| CRP | 278 | rs780094 | -0.0734 | 0.000790892 | 0.381034641 | 0.384743 | 0.17 | 0.0025 | 1.88E-196 | 862.0096 | 0.002541282 |
| CRP | 279 | rs78038982 | 0.0609 | -0.00322226 | 0.017091099 | 0.982463 | 0.14 | 0.0092 | 3.78E-11 | 43.81864367 | 0.000124609 |
| CRP | 280 | rs78248443 | 0.0363 | 0.000313115 | 0.047297819 | 0.953514 | 0.82 | 0.0057 | 1.93E-10 | 40.5567867 | 0.000118752 |
| CRP | 281 | rs78494072 | -0.0498 | 0.00177092 | 0.02647568 | 0.97406 | 0.32 | 0.0076 | 4.53E-11 | 42.93698061 | 0.000127845 |
| CRP | 282 | rs79018068 | -0.0413 | 0.00223849 | 0.034421322 | 0.965732 | 0.15 | 0.0065 | 2.16E-10 | 40.37136095 | 0.000113382 |
| CRP | 283 | rs79219014 | -0.0847 | 0.00106757 | 0.027236149 | 0.972908 | 0.54 | 0.0075 | 7.34E-30 | 127.5393778 | 0.000380146 |
| CRP | 284 | rs79429216 | -0.0769 | 0.00256154 | 0.012359157 | 0.987633 | 0.32 | 0.0106 | 3.20E-13 | 52.63091848 | 0.000144368 |
| CRP | 285 | rs79468673 | 0.0556 | -0.00144414 | 0.034749483 | 0.964655 | 0.35 | 0.0066 | 2.77E-17 | 70.96786042 | 0.000207381 |
| CRP | 286 | rs79634415 | -0.026 | 0.000718958 | 0.088161986 | 0.911527 | 0.47 | 0.0042 | 4.17E-10 | 38.32199546 | 0.000108687 |
| CRP | 287 | rs7976660 | 0.0209 | 0.000391066 | 0.269084996 | 0.729925 | 0.54 | 0.0027 | 6.21E-15 | 59.91906722 | 0.000171822 |
| CRP | 288 | rs80051818 | -0.0401 | 5.41E-05 | 0.326054618 | 0.67824 | 0.93 | 0.0026 | 4.62E-55 | 237.8713018 | 0.000706698 |
| CRP | 289 | rs80255322 | 0.0707 | 0.0025388 | 0.036670209 | 0.963647 | 0.0929994 | 0.0064 | 3.71E-28 | 122.0334473 | 0.000353148 |
| CRP | 290 | rs80257887 | -0.0476 | -0.000293912 | 0.03585206 | 0.964366 | 0.85 | 0.0065 | 2.33E-13 | 53.62745562 | 0.00015664 |
| CRP | 291 | rs80341247 | 0.0397 | 0.00084427 | 0.09516357 | 0.905205 | 0.38 | 0.0041 | 6.54E-22 | 93.75907198 | 0.000271426 |
| CRP | 292 | rs8060025 | -0.0187 | 0.00124454 | 0.389349 | 0.388083 | 0.032 | 0.0024 | 1.59E-14 | 60.71006944 | 0.000166282 |
| CRP | 293 | rs8180849 | -0.0226 | 0.000963304 | 0.13288036 | 0.86628 | 0.25 | 0.0035 | 1.28E-10 | 41.69469388 | 0.000117703 |
| CRP | 294 | rs862994 | -0.0353 | -1.10E-06 | 0.268826573 | 0.268374 | 1 | 0.0027 | 1.73E-39 | 170.9314129 | 0.00048986 |
| CRP | 295 | rs880632 | -0.0171 | -0.00156626 | 0.294027156 | 0.708716 | 0.012 | 0.0027 | 1.24E-10 | 40.11111111 | 0.000121394 |
| CRP | 296 | rs9521499 | 0.0143 | -0.000813456 | 0.415988 | 0.585492 | 0.16 | 0.0024 | 3.63E-09 | 35.50173611 | 9.93584E-05 |
| CRP | 297 | rs9604045 | -0.0229 | -0.00049612 | 0.264431 | 0.746579 | 0.46 | 0.0029 | 3.85E-15 | 62.35552913 | 0.000204003 |
| CRP | 298 | rs9738365 | 0.0203 | 0.000239977 | 0.266574 | 0.734178 | 0.709999 | 0.0027 | 4.93E-14 | 56.52812071 | 0.000161137 |
| CRP | 299 | rs983309 | 0.0606 | -0.00206429 | 0.118058296 | 0.116705 | 0.0189998 | 0.0037 | 5.16E-61 | 268.2512783 | 0.000764736 |
| CTACK | 1 | rs116303454 | -0.383 | -0.00060654 | 0.9712 | 0.97843 | 0.760001 | 0.0816 | 3.27E-06 | 22.03013865 | 0.008205947 |
| CTACK | 2 | rs135564 | 0.1893 | -0.000449713 | 0.7406 | 0.703947 | 0.47 | 0.0268 | 2.43E-12 | 49.89208343 | 0.013768445 |
| CTACK | 3 | rs145902143 | 0.2838 | -0.00143811 | 0.0378 | 0.047333 | 0.28 | 0.0581 | 1.03E-06 | 23.86011417 | 0.005858844 |
| CTACK | 4 | rs17321950 | -0.5262 | -0.000113357 | 0.0278 | 0.02997 | 0.95 | 0.1122 | 3.46E-06 | 21.99459521 | 0.014966908 |
| CTACK | 5 | rs2070074 | -0.4467 | -0.000389692 | 0.1024 | 0.095636 | 0.69 | 0.0374 | 1.79E-32 | 142.6555592 | 0.036681299 |
| CTACK | 6 | rs3766110 | 0.1287 | -0.0012353 | 0.2326 | 0.231612 | 0.0659994 | 0.0278 | 3.86E-06 | 21.43223694 | 0.005913146 |
| CTACK | 7 | rs55764737 | -0.5313 | 0.0019521 | 0.0229 | 0.037132 | 0.2 | 0.0972 | 4.62E-08 | 29.87769585 | 0.012632349 |
| CTACK | 8 | rs57338032 | -0.1583 | 0.000335614 | 0.1799 | 0.164048 | 0.66 | 0.0317 | 6.23E-07 | 24.93694832 | 0.007394176 |
| CTACK | 9 | rs7333764 | -0.2773 | -0.00242603 | 0.9732 | 0.980146 | 0.23 | 0.0593 | 2.85E-06 | 21.86705778 | 0.004011129 |
| CTACK | 10 | rs76395525 | -0.5277 | -0.00444213 | 0.9851 | 0.986794 | 0.0769999 | 0.1083 | 9.55E-07 | 23.7419986 | 0.00817468 |
| CXCL9 | 1 | rs111607343 | 0.521 | -0.000790013 | 0.9622 | 0.967218 | 0.630001 | 0.1119 | 2.83E-06 | 21.67780341 | 0.019745248 |
| CXCL9 | 2 | rs11177248 | -0.3073 | 0.00258898 | 0.9394 | 0.935231 | 0.025 | 0.067 | 4.45E-06 | 21.03659835 | 0.010751729 |
| CXCL9 | 3 | rs112337562 | 0.37 | 0.00368797 | 0.0169 | 0.011828 | 0.19 | 0.0796 | 2.98E-06 | 21.60614631 | 0.00454902 |
| CXCL9 | 4 | rs117831247 | 0.8334 | -0.00363086 | 0.9811 | 0.988183 | 0.17 | 0.1754 | 2.16E-06 | 22.57604251 | 0.025757996 |
| CXCL9 | 5 | rs139010077 | -0.4322 | 0.00269912 | 0.9891 | 0.986287 | 0.28 | 0.095 | 3.55E-06 | 20.6977108 | 0.004027784 |
| CXCL9 | 6 | rs1796086 | 0.2096 | -0.000763999 | 0.0944 | 0.087273 | 0.450001 | 0.0403 | 2.23E-07 | 27.05032357 | 0.007511401 |
| CXCL9 | 7 | rs41272086 | 0.2226 | -0.000278919 | 0.9145 | 0.894469 | 0.760001 | 0.0415 | 7.43E-08 | 28.77094498 | 0.007748723 |
| CXCL9 | 8 | rs5752128 | 0.1685 | -5.72E-05 | 0.0954 | 0.093982 | 0.95 | 0.0369 | 4.34E-06 | 20.85196936 | 0.004900436 |
| CXCL9 | 9 | rs62562991 | -0.6236 | 0.000663649 | 0.9801 | 0.981065 | 0.75 | 0.126 | 8.40E-07 | 24.49464349 | 0.015169305 |
| CXCL9 | 10 | rs6679677 | -0.162 | -0.00164097 | 0.9085 | 0.899239 | 0.0810009 | 0.0329 | 8.86E-07 | 24.24589573 | 0.004363209 |
| CXCL9 | 11 | rs77086208 | -0.3226 | -0.000939113 | 0.9811 | 0.982467 | 0.67 | 0.0698 | 3.83E-06 | 21.36081806 | 0.003859524 |
| CXCL9 | 12 | rs816960 | 0.1224 | 0.00135885 | 0.7406 | 0.77631 | 0.0490004 | 0.0244 | 5.01E-07 | 25.16420317 | 0.005756341 |
| Eotaxin | 1 | rs11087905 | -0.0941 | 0.000506125 | 0.6581 | 0.65176 | 0.450001 | 0.0189 | 5.48E-07 | 24.7888077 | 0.003984742 |
| Eotaxin | 2 | rs112347425 | -0.158 | 0.000203065 | 0.8926 | 0.900902 | 0.83 | 0.0277 | 8.65E-09 | 32.53528653 | 0.00478636 |
| Eotaxin | 3 | rs12075 | -0.1671 | 0.000244659 | 0.3976 | 0.420271 | 0.67 | 0.0156 | 1.33E-26 | 114.7370562 | 0.01337563 |
| Eotaxin | 4 | rs138125931 | -0.1292 | 0.00196288 | 0.8678 | 0.124442 | 0.0219999 | 0.0254 | 3.59E-07 | 25.87364375 | 0.003830065 |
| Eotaxin | 5 | rs1476670 | 0.1007 | 9.55E-05 | 0.7932 | 0.78907 | 0.89 | 0.0217 | 3.51E-06 | 21.5347321 | 0.003326765 |
| Eotaxin | 6 | rs2024050 | -0.1728 | 0.00194947 | 0.8996 | 0.895891 | 0.0359998 | 0.0303 | 1.10E-08 | 32.52387021 | 0.005393872 |
| Eotaxin | 7 | rs2210755 | 0.1104 | 0.00119383 | 0.0805 | 0.079341 | 0.28 | 0.0242 | 4.85E-06 | 20.81169319 | 0.001804329 |
| Eotaxin | 8 | rs2211994 | -0.0885 | -0.000401414 | 0.7495 | 0.736492 | 0.53 | 0.0177 | 6.08E-07 | 25 | 0.002941006 |
| Eotaxin | 9 | rs2228467 | 0.4163 | 0.00152823 | 0.0696 | 0.061446 | 0.19 | 0.0292 | 2.27E-46 | 203.2577524 | 0.022445111 |
| Eotaxin | 10 | rs2419841 | 0.1277 | -0.00280402 | 0.1193 | 0.103868 | 0.00269998 | 0.0279 | 4.98E-06 | 20.94948677 | 0.003426733 |
| Eotaxin | 11 | rs5746492 | -0.0954 | 0.000374238 | 0.1809 | 0.180822 | 0.61 | 0.0207 | 3.96E-06 | 21.24007561 | 0.002697132 |
| Eotaxin | 12 | rs5754733 | 0.1042 | -0.000720925 | 0.2117 | 0.237636 | 0.29 | 0.0214 | 1.06E-06 | 23.70870818 | 0.003623913 |
| Eotaxin | 13 | rs59808887 | 0.1673 | -0.00122503 | 0.9225 | 0.920866 | 0.25 | 0.0358 | 2.91E-06 | 21.83865204 | 0.004002119 |
| Eotaxin | 14 | rs75426604 | 0.1366 | -0.000857355 | 0.8688 | 0.868253 | 0.31 | 0.0291 | 2.53E-06 | 22.03512004 | 0.004253878 |
| Eotaxin | 15 | rs79722574 | 0.1113 | 0.000311823 | 0.84 | 0.836131 | 0.69 | 0.0228 | 1.06E-06 | 23.82981302 | 0.003329811 |
| Eotaxin | 16 | rs9317045 | -0.1182 | 1.11E-06 | 0.1461 | 0.163462 | 1 | 0.0237 | 5.82E-07 | 24.87357795 | 0.003485958 |
| GROa | 1 | rs1113500 | -0.1174 | 0.000102069 | 0.3698 | 0.365454 | 0.86 | 0.0244 | 1.57E-06 | 23.15029562 | 0.006424088 |
| GROa | 2 | rs118158560 | -0.2703 | -0.000482289 | 0.9374 | 0.941182 | 0.69 | 0.0594 | 3.42E-06 | 20.70709621 | 0.008574748 |
| GROa | 3 | rs12075 | -0.3751 | 0.000244659 | 0.3976 | 0.420271 | 0.67 | 0.0237 | 1.24E-55 | 250.4940626 | 0.067399312 |
| GROa | 4 | rs140734053 | -0.7257 | 0.00363953 | 0.9751 | 0.982381 | 0.0949992 | 0.1561 | 3.58E-06 | 21.61267088 | 0.025573652 |
| GROa | 5 | rs188345231 | -0.623 | -0.000463199 | 0.9881 | 0.980403 | 0.82 | 0.1323 | 4.34E-06 | 22.17463117 | 0.009127544 |
| GROa | 6 | rs508977 | 0.3802 | -0.000520497 | 0.2336 | 0.239167 | 0.43 | 0.028 | 7.56E-42 | 184.377602 | 0.051758604 |
| GROa | 7 | rs62024303 | 0.3053 | -0.00223059 | 0.0457 | 0.043287 | 0.11 | 0.0666 | 4.41E-06 | 21.01382689 | 0.008129891 |
| GROa | 8 | rs78653452 | 0.7362 | 0.00114746 | 0.9881 | 0.987171 | 0.649999 | 0.1558 | 1.21E-06 | 22.32835454 | 0.01274587 |
| IFNg | 1 | rs113600793 | -0.1829 | 0.00215742 | 0.9602 | 0.94912 | 0.15 | 0.0373 | 8.95E-07 | 24.04416764 | 0.002556832 |
| IFNg | 2 | rs115729819 | -0.2484 | -0.00140335 | 0.0209 | 0.014649 | 0.55 | 0.0515 | 1.38E-06 | 23.26423226 | 0.002525262 |
| IFNg | 3 | rs11843756 | -0.184 | -7.46E-05 | 0.0288 | 0.027023 | 0.97 | 0.0393 | 3.09E-06 | 21.9205045 | 0.001893943 |
| IFNg | 4 | rs12420286 | -0.2376 | 0.000748826 | 0.0288 | 0.042581 | 0.59 | 0.0501 | 2.08E-06 | 22.49144824 | 0.003158087 |
| IFNg | 5 | rs1867282 | -0.0774 | -0.000306388 | 0.674 | 0.682727 | 0.62 | 0.0166 | 3.15E-06 | 21.74031064 | 0.002632628 |
| IFNg | 6 | rs2073438 | -0.0898 | 0.00080715 | 0.7286 | 0.703402 | 0.2 | 0.0188 | 1.68E-06 | 22.81586691 | 0.003189199 |
| IFNg | 7 | rs4730203 | 0.0983 | 0.00171841 | 0.8618 | 0.872983 | 0.0439997 | 0.0203 | 1.32E-06 | 23.44849426 | 0.002301715 |
| IFNg | 8 | rs6902031 | 0.1166 | 0.0019336 | 0.8877 | 0.924888 | 0.0719996 | 0.0242 | 1.41E-06 | 23.21487603 | 0.002710648 |
| IFNg | 9 | rs7073753 | -0.0821 | -0.000729189 | 0.5666 | 0.5825 | 0.2 | 0.0167 | 8.39E-07 | 24.16870451 | 0.00331041 |
| IFNg | 10 | rs74148555 | 0.3732 | -0.000383461 | 0.9473 | 0.969336 | 0.82 | 0.0774 | 2.64E-06 | 23.24884322 | 0.013906294 |
| IFNg | 11 | rs78296352 | -0.343 | 0.000744327 | 0.9712 | 0.961221 | 0.61 | 0.0652 | 1.38E-07 | 27.67534533 | 0.006581417 |
| IL2 | 1 | rs12051139 | 0.1131 | -4.45E-05 | 0.4006 | 0.409894 | 0.94 | 0.0247 | 4.76E-06 | 20.966759 | 0.006143034 |
| IL2 | 2 | rs13412535 | -0.1764 | -0.000554013 | 0.7744 | 0.7682 | 0.41 | 0.0332 | 1.18E-07 | 28.23065757 | 0.010872555 |
| IL2 | 3 | rs1534019 | 0.1187 | 1.06E-05 | 0.5557 | 0.56268 | 0.99 | 0.0248 | 1.58E-06 | 22.90857505 | 0.006957419 |
| IL2 | 4 | rs170117 | 0.1617 | 0.000702633 | 0.8797 | 0.870478 | 0.41 | 0.0349 | 3.87E-06 | 21.46689272 | 0.005534141 |
| IL2 | 5 | rs2807544 | -0.1175 | -0.000900872 | 0.5656 | 0.550653 | 0.12 | 0.0253 | 3.41E-06 | 21.56923245 | 0.006784298 |
| IL2 | 6 | rs4634519 | 0.1261 | 0.000433784 | 0.3121 | 0.28548 | 0.49 | 0.0269 | 2.77E-06 | 21.97483451 | 0.006827776 |
| IL2 | 7 | rs61335305 | -0.4514 | 0.00117417 | 0.9891 | 0.981842 | 0.58 | 0.0918 | 7.32E-07 | 24.17896725 | 0.004393593 |
| IL2 | 8 | rs62124990 | 0.6961 | 0.00323045 | 0.9841 | 0.973578 | 0.0719996 | 0.1495 | 3.22E-06 | 21.68008009 | 0.015163855 |
| IL2 | 9 | rs7615304 | 0.1172 | 0.000163943 | 0.6511 | 0.641128 | 0.780001 | 0.0242 | 1.21E-06 | 23.45440885 | 0.006240708 |
| IL2 | 10 | rs80336398 | -0.4001 | -0.000630055 | 0.0209 | 0.014696 | 0.8 | 0.0858 | 2.82E-06 | 21.74515597 | 0.006551495 |
| IL6 | 1 | rs114373846 | -0.422 | -0.00157775 | 0.9871 | 0.984095 | 0.51 | 0.0904 | 3.32E-06 | 21.79154593 | 0.004535297 |
| IL6 | 2 | rs12024374 | -0.1096 | 0.00131452 | 0.8837 | 0.922069 | 0.21 | 0.0236 | 3.35E-06 | 21.5673657 | 0.002469083 |
| IL6 | 3 | rs1333040 | -0.0738 | 0.00070419 | 0.4264 | 0.414034 | 0.22 | 0.0158 | 3.17E-06 | 21.81717673 | 0.002664214 |
| IL6 | 4 | rs13412535 | 0.1164 | -0.000554013 | 0.7744 | 0.7682 | 0.41 | 0.0215 | 7.34E-08 | 29.31089237 | 0.004734132 |
| IL6 | 5 | rs72831623 | -0.1973 | 0.00285112 | 0.9473 | 0.937638 | 0.0340001 | 0.0372 | 1.08E-07 | 28.12990664 | 0.003886712 |
| IL6 | 6 | rs73273528 | -0.2672 | -0.00202577 | 0.9662 | 0.964732 | 0.19 | 0.0553 | 9.58E-07 | 23.34654637 | 0.004663228 |
| IL6 | 7 | rs76856708 | -0.3289 | -0.000944101 | 0.0378 | 0.03797 | 0.53 | 0.07 | 2.61E-06 | 22.07657347 | 0.007868916 |
| IL7 | 1 | rs117509142 | 0.327 | 0.000930161 | 0.0567 | 0.043253 | 0.54 | 0.0688 | 1.99E-06 | 22.59012473 | 0.011438219 |
| IL7 | 2 | rs141425475 | 0.4781 | 0.000478213 | 0.0288 | 0.027004 | 0.79 | 0.1016 | 2.53E-06 | 22.14369401 | 0.012786999 |
| IL7 | 3 | rs144701438 | 0.4819 | 0.00134597 | 0.9462 | 0.966094 | 0.42 | 0.0989 | 9.75E-07 | 23.74221696 | 0.023643353 |
| IL7 | 4 | rs17091524 | -0.4924 | -0.000496497 | 0.0427 | 0.031563 | 0.77 | 0.1013 | 1.91E-06 | 23.62746877 | 0.019821751 |
| IL7 | 5 | rs218260 | 0.1337 | -0.00065656 | 0.7883 | 0.780791 | 0.34 | 0.0286 | 2.93E-06 | 21.85399042 | 0.005966301 |
| IL7 | 6 | rs28793375 | -0.1638 | -0.00117726 | 0.833 | 0.870202 | 0.16 | 0.0361 | 4.46E-06 | 20.58796357 | 0.007464819 |
| IL7 | 7 | rs4320361 | 0.3245 | 0.000519369 | 0.5477 | 0.522506 | 0.37 | 0.0249 | 6.87E-39 | 169.8363736 | 0.052170948 |
| IL7 | 8 | rs62006410 | 0.1557 | 0.000745222 | 0.7853 | 0.765651 | 0.3 | 0.0303 | 3.39E-07 | 26.40535242 | 0.008174757 |
| IL7 | 9 | rs75904417 | 0.1698 | 7.44E-06 | 0.1412 | 0.109299 | 0.99 | 0.0349 | 1.16E-06 | 23.67143127 | 0.006992494 |
| IL7 | 10 | rs77318030 | 0.2921 | -0.0021177 | 0.0517 | 0.056293 | 0.0949992 | 0.0632 | 3.74E-06 | 21.36136286 | 0.008366222 |
| IL7 | 11 | rs77981494 | 0.5178 | 0.00054054 | 0.0159 | 0.020643 | 0.79 | 0.1064 | 1.07E-06 | 23.68322474 | 0.00839055 |
| IL8 | 1 | rs11634944 | 0.1214 | 0.000782113 | 0.3469 | 0.3661 | 0.19 | 0.0252 | 1.29E-06 | 23.20792391 | 0.006678076 |
| IL8 | 2 | rs12075 | -0.12 | 0.000244659 | 0.3976 | 0.420271 | 0.67 | 0.0236 | 3.88E-07 | 25.85463947 | 0.00689801 |
| IL8 | 3 | rs141926526 | 0.6149 | -0.000193323 | 0.0477 | 0.039721 | 0.89 | 0.1308 | 2.57E-06 | 22.10006231 | 0.034350348 |
| IL8 | 4 | rs2673604 | 0.1266 | 0.000277583 | 0.2992 | 0.281843 | 0.66 | 0.0255 | 7.02E-07 | 24.6483045 | 0.006721297 |
| IL10 | 1 | rs10457128 | 0.0865 | 0.000608637 | 0.3608 | 0.372492 | 0.3 | 0.0172 | 5.24E-07 | 25.29154273 | 0.003451163 |
| IL10 | 2 | rs10493718 | 0.11 | 0.000218471 | 0.7087 | 0.774223 | 0.75 | 0.0222 | 7.16E-07 | 24.55157861 | 0.004995952 |
| IL10 | 3 | rs10809307 | -0.1305 | -0.00122963 | 0.6958 | 0.689396 | 0.0460002 | 0.0282 | 3.64E-06 | 21.41523314 | 0.007209326 |
| IL10 | 4 | rs11206302 | 0.1189 | -0.00113881 | 0.0795 | 0.078535 | 0.28 | 0.0251 | 2.20E-06 | 22.43965969 | 0.002069115 |
| IL10 | 5 | rs113831257 | -0.3592 | 0.00100883 | 0.9583 | 0.958028 | 0.49 | 0.0644 | 2.53E-08 | 31.1100652 | 0.010311936 |
| IL10 | 6 | rs11626201 | -0.1162 | -0.000382321 | 0.3658 | 0.407844 | 0.51 | 0.0245 | 1.93E-06 | 22.49469388 | 0.006264872 |
| IL10 | 7 | rs143799975 | 0.7984 | 0.00130167 | 0.0129 | 0.011827 | 0.62 | 0.1637 | 1.00E-06 | 23.78722047 | 0.016233864 |
| IL10 | 8 | rs2086656 | 0.0789 | -0.000613788 | 0.3052 | 0.296368 | 0.32 | 0.0171 | 3.78E-06 | 21.28931979 | 0.002640148 |
| IL10 | 9 | rs282258 | -0.0992 | 7.26E-05 | 0.5746 | 0.569493 | 0.9 | 0.0162 | 1.00E-09 | 37.49672306 | 0.004810791 |
| IL10 | 10 | rs3025021 | -0.0947 | 4.09E-05 | 0.6322 | 0.666093 | 0.95 | 0.0195 | 1.46E-06 | 23.58472058 | 0.004170577 |
| IL10 | 11 | rs34383175 | 0.3153 | -0.00266345 | 0.9732 | 0.964356 | 0.0810009 | 0.0657 | 1.51E-06 | 23.03123371 | 0.005185789 |
| IL10 | 12 | rs35794877 | -0.0794 | 0.000683034 | 0.508 | 0.485792 | 0.23 | 0.0167 | 1.80E-06 | 22.60518484 | 0.003151373 |
| IL10 | 13 | rs397816 | -0.1237 | 0.000178094 | 0.4264 | 0.444625 | 0.760001 | 0.0249 | 7.90E-07 | 24.6797471 | 0.007485068 |
| IL10 | 14 | rs41282660 | 0.1194 | -0.00102472 | 0.1322 | 0.115464 | 0.25 | 0.0255 | 3.72E-06 | 21.92442907 | 0.003271069 |
| IL10 | 15 | rs4349809 | -0.2853 | -0.000580593 | 0.4513 | 0.477342 | 0.31 | 0.0165 | 5.77E-67 | 298.9755372 | 0.040311952 |
| IL10 | 16 | rs465757 | -0.084 | 0.000201096 | 0.333 | 0.318093 | 0.74 | 0.0174 | 1.17E-06 | 23.30558859 | 0.00313443 |
| IL10 | 17 | rs4741748 | 0.0793 | -0.00118249 | 0.4284 | 0.445307 | 0.0389996 | 0.017 | 2.79E-06 | 21.75948097 | 0.003079768 |
| IL10 | 18 | rs7088799 | 0.0852 | 0.000734108 | 0.4344 | 0.417519 | 0.2 | 0.0167 | 3.23E-07 | 26.02832658 | 0.003567043 |
| IL10 | 19 | rs7645625 | 0.1086 | -0.000424828 | 0.4324 | 0.431584 | 0.46 | 0.0237 | 4.41E-06 | 20.99727608 | 0.005789189 |
| IL10 | 20 | rs79848609 | -0.2603 | -0.00132088 | 0.0318 | 0.047865 | 0.33 | 0.0537 | 8.75E-07 | 23.49631548 | 0.004172252 |
| IL10 | 21 | rs8112909 | 0.1426 | -0.000501195 | 0.1918 | 0.202008 | 0.48 | 0.0299 | 1.94E-06 | 22.74556213 | 0.006304295 |
| IL10 | 22 | rs9450351 | 0.2768 | 0.000658307 | 0.0616 | 0.073784 | 0.57 | 0.0489 | 1.48E-08 | 32.0416191 | 0.008857902 |
| IL13 | 1 | rs117795020 | 0.3522 | 0.00137729 | 0.9821 | 0.981729 | 0.52 | 0.0716 | 9.86E-07 | 24.19650604 | 0.004361315 |
| IL13 | 2 | rs12623722 | 0.1185 | 0.000411704 | 0.7177 | 0.705695 | 0.51 | 0.0258 | 4.19E-06 | 21.09586263 | 0.005690108 |
| IL13 | 3 | rs139083458 | -0.9902 | -0.00163226 | 0.9841 | 0.982127 | 0.450001 | 0.2107 | 2.81E-06 | 22.08598499 | 0.030684016 |
| IL13 | 4 | rs142167313 | 0.313 | 0.00195215 | 0.0189 | 0.010313 | 0.51 | 0.0617 | 3.98E-07 | 25.7346548 | 0.003633237 |
| IL13 | 5 | rs27949 | 0.1168 | -1.38E-05 | 0.3688 | 0.318717 | 0.98 | 0.0252 | 3.43E-06 | 21.48248929 | 0.00635146 |
| IL13 | 6 | rs6799107 | 0.1459 | -4.32E-05 | 0.2137 | 0.20818 | 0.95 | 0.0301 | 1.25E-06 | 23.49511595 | 0.007153744 |
| IL13 | 7 | rs7073807 | -0.1682 | 0.00141151 | 0.8827 | 0.887944 | 0.12 | 0.0356 | 2.37E-06 | 22.32297058 | 0.00585859 |
| IL13 | 8 | rs75995699 | -0.3319 | 0.000180444 | 0.9761 | 0.975187 | 0.92 | 0.0698 | 2.64E-06 | 22.61016125 | 0.005139688 |
| IL13 | 9 | rs9472168 | -0.4244 | -0.000520958 | 0.4493 | 0.477474 | 0.37 | 0.0248 | 1.08E-65 | 292.8514568 | 0.089131711 |
| IL14 | 1 | rs10512267 | 0.0824 | 0.000157145 | 0.33 | 0.330807 | 0.8 | 0.0161 | 2.94E-07 | 26.19405116 | 0.003002432 |
| IL14 | 2 | rs116705532 | 0.4678 | 0.00428499 | 0.0169 | 0.016915 | 0.0549997 | 0.0978 | 1.76E-06 | 22.8792996 | 0.007271681 |
| IL14 | 3 | rs117146485 | 0.2924 | 0.000520791 | 0.0149 | 0.009915 | 0.86 | 0.0629 | 2.71E-06 | 21.60993426 | 0.002509871 |
| IL14 | 4 | rs17713451 | -0.1274 | -0.00151057 | 0.835 | 0.862671 | 0.0669993 | 0.0253 | 4.97E-07 | 25.35699667 | 0.004472386 |
| IL14 | 5 | rs2332982 | -0.1118 | 0.000345816 | 0.1421 | 0.172673 | 0.649999 | 0.0219 | 3.20E-07 | 26.06125811 | 0.003047504 |
| IL14 | 6 | rs6765768 | -0.0795 | -0.000225433 | 0.3917 | 0.37882 | 0.7 | 0.0167 | 2.00E-06 | 22.66216071 | 0.003011866 |
| IL14 | 7 | rs73023729 | 0.1796 | 0.0046447 | 0.9771 | 0.984079 | 0.04 | 0.0366 | 9.03E-07 | 24.07966795 | 0.001443501 |
| IL14 | 8 | rs7613691 | -0.1775 | -0.000940762 | 0.0527 | 0.062848 | 0.42 | 0.0384 | 4.05E-06 | 21.36654324 | 0.003145755 |
| IL14 | 9 | rs79597994 | 0.5831 | -0.000322827 | 0.9722 | 0.972918 | 0.86 | 0.127 | 4.32E-06 | 21.08038998 | 0.018378772 |
| IL14 | 10 | rs9508291 | 0.1676 | 0.00052551 | 0.0646 | 0.066391 | 0.64 | 0.0359 | 3.03E-06 | 21.79511332 | 0.003394751 |
| IL14 | 11 | rs9941733 | -0.114 | 0.000633643 | 0.175 | 0.170816 | 0.4 | 0.0229 | 6.88E-07 | 24.78213611 | 0.003752595 |
| IL6 | 1 | rs116135478 | -0.8206 | 0.000505716 | 0.0179 | 0.020653 | 0.8 | 0.166 | 3.66E-06 | 24.4369415 | 0.023675642 |
| IL6 | 2 | rs117217798 | 0.2036 | -0.00147888 | 0.9314 | 0.932061 | 0.2 | 0.0444 | 4.15E-06 | 21.02759516 | 0.005297194 |
| IL6 | 3 | rs117916513 | 0.502 | 4.50E-05 | 0.9791 | 0.987112 | 0.99 | 0.0986 | 3.79E-07 | 25.92111056 | 0.010313611 |
| IL6 | 4 | rs1255143 | -0.1306 | 0.000368784 | 0.4344 | 0.460263 | 0.52 | 0.0242 | 7.10E-08 | 29.12430845 | 0.008381381 |
| IL6 | 5 | rs12765671 | 0.6023 | -0.000193727 | 0.9831 | 0.973868 | 0.91 | 0.1318 | 4.84E-06 | 20.883097 | 0.012054248 |
| IL6 | 6 | rs144691581 | -0.4882 | 0.00563311 | 0.9851 | 0.98487 | 0.02 | 0.0967 | 4.20E-07 | 25.48840164 | 0.006996682 |
| IL6 | 7 | rs1801020 | -0.1733 | 0.000732165 | 0.7753 | 0.745565 | 0.26 | 0.0272 | 4.53E-10 | 40.59376352 | 0.010464054 |
| IL6 | 8 | rs4253283 | -0.146 | 0.000119989 | 0.6998 | 0.683836 | 0.84 | 0.0262 | 1.75E-08 | 31.05296894 | 0.008956129 |
| IL6 | 9 | rs4513633 | 0.2239 | 0.000890663 | 0.1243 | 0.13345 | 0.29 | 0.0453 | 7.44E-07 | 24.42934277 | 0.010913515 |
| IL6 | 10 | rs4778636 | 0.7272 | -4.49E-05 | 0.9254 | 0.90814 | 0.96 | 0.0633 | 1.11E-30 | 131.9776285 | 0.073013986 |
| IL7 | 1 | rs117556572 | 0.5102 | 4.88E-05 | 0.9891 | 0.98268 | 0.98 | 0.1099 | 3.28E-06 | 21.55189804 | 0.005612775 |
| IL7 | 2 | rs1530455 | -0.108 | 0.00103628 | 0.5915 | 0.595432 | 0.0759994 | 0.0173 | 4.87E-10 | 38.97223429 | 0.005636692 |
| IL7 | 3 | rs17106604 | -0.1129 | -0.00128213 | 0.8757 | 0.888608 | 0.15 | 0.0225 | 6.37E-07 | 25.17809383 | 0.002774881 |
| IL7 | 4 | rs17282552 | 0.2001 | -0.000208212 | 0.0268 | 0.032361 | 0.9 | 0.0405 | 8.21E-07 | 24.41091907 | 0.002088628 |
| IL7 | 5 | rs187475560 | 0.2434 | -0.000421959 | 0.9881 | 0.989828 | 0.89 | 0.052 | 3.29E-06 | 21.90960059 | 0.001393218 |
| IL7 | 6 | rs34120897 | 0.1055 | 0.000362422 | 0.0974 | 0.117121 | 0.68 | 0.0232 | 3.63E-06 | 20.67897221 | 0.001956993 |
| IL7 | 7 | rs62191444 | 0.1136 | -0.000751481 | 0.834 | 0.83528 | 0.33 | 0.0247 | 4.22E-06 | 21.15255126 | 0.003573229 |
| IL7 | 8 | rs78296352 | -0.3027 | 0.000744327 | 0.9712 | 0.961221 | 0.61 | 0.0646 | 4.27E-06 | 21.95633285 | 0.005125733 |
| IL7 | 9 | rs78612928 | -0.1037 | -0.000120516 | 0.1968 | 0.189953 | 0.87 | 0.0222 | 2.62E-06 | 21.81984011 | 0.003399666 |
| IL7 | 10 | rs79462337 | -0.2097 | -0.00469511 | 0.0199 | 0.020487 | 0.0309999 | 0.0435 | 1.41E-06 | 23.23904875 | 0.00171534 |
| IL8 | 1 | rs116383510 | 0.5426 | -0.00303232 | 0.0199 | 0.013902 | 0.22 | 0.1056 | 3.00E-07 | 26.40169091 | 0.011484525 |
| IL8 | 2 | rs11700536 | -0.1156 | 0.0010081 | 0.6382 | 0.593636 | 0.0819993 | 0.025 | 4.21E-06 | 21.381376 | 0.00617122 |
| IL8 | 3 | rs117266781 | -0.6841 | -0.000763125 | 0.9891 | 0.987836 | 0.77 | 0.1468 | 3.15E-06 | 21.71636186 | 0.010091039 |
| IL8 | 4 | rs17229943 | 0.312 | 0.00159126 | 0.0497 | 0.050526 | 0.22 | 0.0463 | 1.62E-11 | 45.40955082 | 0.009195097 |
| IL8 | 5 | rs1852105 | -0.3036 | 0.00109163 | 0.9513 | 0.943599 | 0.37 | 0.0661 | 4.32E-06 | 21.09602422 | 0.008540435 |
| IL8 | 6 | rs1979967 | -0.1402 | -0.00104938 | 0.7406 | 0.767741 | 0.12 | 0.0286 | 9.45E-07 | 24.03056384 | 0.007552308 |
| IL8 | 7 | rs2729385 | -0.1231 | -0.000874964 | 0.675 | 0.688595 | 0.15 | 0.0262 | 3.79E-06 | 22.07565119 | 0.006648646 |
| IL8 | 8 | rs385076 | 0.2432 | 0.00019479 | 0.6471 | 0.644264 | 0.74 | 0.0248 | 1.66E-22 | 96.16649324 | 0.027013459 |
| IL8 | 9 | rs4482818 | -0.1286 | 0.000943939 | 0.3588 | 0.368874 | 0.11 | 0.0244 | 1.45E-07 | 27.77808385 | 0.007609531 |
| IL8 | 10 | rs658805 | -0.1226 | -0.000584707 | 0.6869 | 0.671042 | 0.33 | 0.0244 | 4.94E-07 | 25.24650632 | 0.006465283 |
| IL8 | 11 | rs71478720 | 0.2669 | 0.000905893 | 0.7823 | 0.742892 | 0.16 | 0.0276 | 3.71E-22 | 93.51450588 | 0.024263805 |
| IL8 | 12 | rs78623212 | -0.8705 | 0.00235917 | 0.9761 | 0.975274 | 0.2 | 0.1778 | 6.71E-07 | 23.97033136 | 0.035355726 |
| IL8 | 13 | rs78716465 | -0.3265 | -0.0031007 | 0.9622 | 0.960401 | 0.0369999 | 0.0682 | 1.63E-06 | 22.91910329 | 0.007754495 |
| IL1ra | 1 | rs1054402 | -0.1311 | 6.91E-05 | 0.7336 | 0.751215 | 0.92 | 0.027 | 1.13E-06 | 23.57641975 | 0.006717829 |
| IL1ra | 2 | rs11627423 | -0.1171 | -0.000642629 | 0.3897 | 0.40766 | 0.27 | 0.0247 | 2.12E-06 | 22.47604452 | 0.006522552 |
| IL1ra | 3 | rs12121840 | -0.2692 | 0.000161969 | 0.9155 | 0.935717 | 0.89 | 0.0571 | 2.43E-06 | 22.22684877 | 0.011212312 |
| IL1ra | 4 | rs2809154 | 0.1786 | -0.00031229 | 0.838 | 0.815038 | 0.67 | 0.0388 | 3.74E-06 | 21.18846317 | 0.008660679 |
| IL1ra | 5 | rs61335305 | -0.4453 | 0.00117417 | 0.9891 | 0.981842 | 0.58 | 0.0908 | 1.00E-06 | 24.05103073 | 0.004275649 |
| IL1ra | 6 | rs9623661 | 0.1966 | 0.000195773 | 0.9076 | 0.907627 | 0.84 | 0.0426 | 3.86E-06 | 21.29844167 | 0.006482813 |
| IL2ra | 1 | rs11241559 | 0.1264 | 8.72E-05 | 0.7813 | 0.766394 | 0.9 | 0.0266 | 2.00E-06 | 22.58036068 | 0.005459976 |
| IL2ra | 2 | rs117244812 | 0.7064 | 0.00370337 | 0.9861 | 0.988037 | 0.17 | 0.1488 | 2.10E-06 | 22.53696959 | 0.013679403 |
| IL2ra | 3 | rs12722497 | -0.6279 | -0.00033753 | 0.8559 | 0.910015 | 0.74 | 0.0485 | 1.57E-38 | 167.6090594 | 0.097251872 |
| IL2ra | 4 | rs185231391 | -0.8503 | -0.00127646 | 0.0149 | 0.012817 | 0.62 | 0.1809 | 1.47E-06 | 22.09363752 | 0.02122467 |
| IL2ra | 5 | rs4733117 | -0.1369 | -0.000499535 | 0.164 | 0.145281 | 0.54 | 0.0292 | 2.63E-06 | 21.98068352 | 0.005139099 |
| IL2ra | 6 | rs61705228 | -0.3303 | -0.0020539 | 0.9632 | 0.944929 | 0.1 | 0.0716 | 3.99E-06 | 21.28095448 | 0.007734129 |
| IL2ra | 7 | rs62135626 | 0.1661 | -0.000171903 | 0.8847 | 0.861853 | 0.83 | 0.035 | 1.99E-06 | 22.52180408 | 0.005628525 |
| MCP1 | 1 | rs10145849 | 0.0755 | -0.000293422 | 0.6511 | 0.626125 | 0.61 | 0.0162 | 3.41E-06 | 21.72020271 | 0.002589838 |
| MCP1 | 2 | rs10744620 | -0.0788 | 0.000929185 | 0.6392 | 0.610861 | 0.11 | 0.0161 | 9.91E-07 | 23.95524864 | 0.002864084 |
| MCP1 | 3 | rs111995966 | -0.1452 | 0.000122873 | 0.0258 | 0.019981 | 0.95 | 0.031 | 2.53E-06 | 21.93864724 | 0.001059817 |
| MCP1 | 4 | rs12073356 | 0.1426 | 0.0030245 | 0.9145 | 0.936281 | 0.00909997 | 0.0311 | 4.17E-06 | 21.0241416 | 0.00317994 |
| MCP1 | 5 | rs12075 | -0.2185 | 0.000244659 | 0.3976 | 0.420271 | 0.67 | 0.0155 | 1.44E-44 | 198.7190427 | 0.022869897 |
| MCP1 | 6 | rs146522229 | 0.5976 | -0.00038237 | 0.9761 | 0.987561 | 0.88 | 0.1177 | 3.56E-07 | 25.77912972 | 0.016662624 |
| MCP1 | 7 | rs2036297 | -0.119 | 0.000971876 | 0.6571 | 0.647594 | 0.1 | 0.016 | 1.09E-13 | 55.31640625 | 0.006381501 |
| MCP1 | 8 | rs2288370 | 0.1031 | 0.00119407 | 0.3956 | 0.371593 | 0.0420001 | 0.0163 | 2.25E-10 | 40.00756521 | 0.005083093 |
| MCP1 | 9 | rs2712431 | 0.0787 | -0.000256213 | 0.2853 | 0.314552 | 0.67 | 0.0172 | 4.76E-06 | 20.93594511 | 0.002525835 |
| MCP1 | 10 | rs56212190 | -0.181 | -0.00129303 | 0.9453 | 0.954453 | 0.35 | 0.0373 | 9.85E-07 | 23.54721158 | 0.003388006 |
| MCP1 | 11 | rs7197349 | -0.0968 | 0.000250589 | 0.1372 | 0.127011 | 0.77 | 0.0206 | 2.62E-06 | 22.08087473 | 0.002218426 |
| MCP1 | 12 | rs7632755 | -0.2938 | -0.00102828 | 0.9195 | 0.929977 | 0.36 | 0.0316 | 1.18E-20 | 86.4429178 | 0.012778539 |
| MCP1 | 13 | rs9317045 | -0.1134 | 1.11E-06 | 0.1461 | 0.163462 | 1 | 0.0236 | 1.52E-06 | 23.08883941 | 0.003208583 |
| MIF | 1 | rs118055855 | -0.6907 | 0.00255523 | 0.0129 | 0.015513 | 0.27 | 0.15 | 4.13E-06 | 21.20295511 | 0.012149538 |
| MIF | 2 | rs12594190 | -0.1355 | 0.00106366 | 0.2465 | 0.222292 | 0.12 | 0.0267 | 3.70E-07 | 25.75467463 | 0.006820383 |
| MIF | 3 | rs13142904 | 0.223 | -0.000569676 | 0.9314 | 0.930839 | 0.62 | 0.0425 | 2.56E-07 | 27.5316263 | 0.006354773 |
| MIF | 4 | rs141009259 | 0.6178 | 0.00253489 | 0.0129 | 0.01171 | 0.35 | 0.1322 | 2.47E-06 | 21.83900751 | 0.009720233 |
| MIF | 5 | rs5751777 | 0.1342 | -0.000109999 | 0.3887 | 0.413761 | 0.85 | 0.0249 | 7.09E-08 | 29.04733795 | 0.008558624 |
| MIF | 6 | rs78098071 | 0.4867 | -0.0028985 | 0.0189 | 0.011882 | 0.27 | 0.0918 | 1.78E-07 | 28.10847798 | 0.008784717 |
| MIG | 1 | rs111607343 | 0.521 | -0.000790013 | 0.9622 | 0.967218 | 0.630001 | 0.1119 | 2.83E-06 | 21.67780341 | 0.019745248 |
| MIG | 2 | rs11177248 | -0.3073 | 0.00258898 | 0.9394 | 0.935231 | 0.025 | 0.067 | 4.45E-06 | 21.03659835 | 0.010751729 |
| MIG | 3 | rs112337562 | 0.37 | 0.00368797 | 0.0169 | 0.011828 | 0.19 | 0.0796 | 2.98E-06 | 21.60614631 | 0.00454902 |
| MIG | 4 | rs117831247 | 0.8334 | -0.00363086 | 0.9811 | 0.988183 | 0.17 | 0.1754 | 2.16E-06 | 22.57604251 | 0.025757996 |
| MIG | 5 | rs139010077 | -0.4322 | 0.00269912 | 0.9891 | 0.986287 | 0.28 | 0.095 | 3.55E-06 | 20.6977108 | 0.004027784 |
| MIG | 6 | rs1796086 | 0.2096 | -0.000763999 | 0.0944 | 0.087273 | 0.450001 | 0.0403 | 2.23E-07 | 27.05032357 | 0.007511401 |
| MIG | 7 | rs41272086 | 0.2226 | -0.000278919 | 0.9145 | 0.894469 | 0.760001 | 0.0415 | 7.43E-08 | 28.77094498 | 0.007748723 |
| MIG | 8 | rs5752128 | 0.1685 | -5.72E-05 | 0.0954 | 0.093982 | 0.95 | 0.0369 | 4.34E-06 | 20.85196936 | 0.004900436 |
| MIG | 9 | rs62562991 | -0.6236 | 0.000663649 | 0.9801 | 0.981065 | 0.75 | 0.126 | 8.40E-07 | 24.49464349 | 0.015169305 |
| MIG | 10 | rs6679677 | -0.162 | -0.00164097 | 0.9085 | 0.899239 | 0.0810009 | 0.0329 | 8.86E-07 | 24.24589573 | 0.004363209 |
| MIG | 11 | rs77086208 | -0.3226 | -0.000939113 | 0.9811 | 0.982467 | 0.67 | 0.0698 | 3.83E-06 | 21.36081806 | 0.003859524 |
| MIG | 12 | rs816960 | 0.1224 | 0.00135885 | 0.7406 | 0.77631 | 0.0490004 | 0.0244 | 5.01E-07 | 25.16420317 | 0.005756341 |
| MIP1a | 1 | rs10835056 | -0.1194 | -0.00102181 | 0.7555 | 0.740003 | 0.12 | 0.0254 | 2.60E-06 | 22.09740219 | 0.005266863 |
| MIP1a | 2 | rs12690897 | -0.1248 | 4.46E-05 | 0.7187 | 0.738518 | 0.95 | 0.0262 | 2.11E-06 | 22.68958685 | 0.006297621 |
| MIP1a | 3 | rs34771762 | -0.249 | 0.000505363 | 0.0736 | 0.067707 | 0.649999 | 0.0523 | 2.13E-06 | 22.66706638 | 0.008454833 |
| MIP1a | 4 | rs57786342 | -0.1314 | -0.00202761 | 0.7843 | 0.796325 | 0.00409996 | 0.0285 | 4.06E-06 | 21.25695291 | 0.005841886 |
| MIP1a | 5 | rs60198979 | 0.2146 | 5.27E-05 | 0.9135 | 0.914817 | 0.96 | 0.0458 | 2.62E-06 | 21.95474915 | 0.007278034 |
| MIP1a | 6 | rs6900267 | 0.2429 | 0.00141966 | 0.0746 | 0.055515 | 0.28 | 0.0519 | 2.89E-06 | 21.9038428 | 0.008146168 |
| MIP1a | 7 | rs7232268 | -0.2821 | -0.00176859 | 0.9523 | 0.964191 | 0.25 | 0.0599 | 2.55E-06 | 22.17953963 | 0.007229834 |
| MIP1b | 1 | rs113010081 | 0.5954 | -0.00122706 | 0.1083 | 0.116748 | 0.17 | 0.0236 | 3.85E-140 | 636.4930336 | 0.068469141 |
| MIP1b | 2 | rs113877493 | 0.6124 | -0.000796509 | 0.8777 | 0.918681 | 0.44 | 0.0218 | 1.62E-173 | 789.1460315 | 0.08051428 |
| MIP1b | 3 | rs116237296 | -0.5437 | 0.00155196 | 0.9841 | 0.988622 | 0.57 | 0.1115 | 7.23E-07 | 23.77765006 | 0.009250922 |
| MIP1b | 4 | rs117453826 | 0.5774 | -0.00389852 | 0.0159 | 0.016969 | 0.0819993 | 0.0593 | 5.07E-22 | 94.80782257 | 0.010433257 |
| MIP1b | 5 | rs12490293 | -0.0866 | -0.000253622 | 0.3917 | 0.42807 | 0.66 | 0.0168 | 2.12E-07 | 26.57157029 | 0.003573857 |
| MIP1b | 6 | rs141102180 | -0.3225 | -0.000924348 | 0.9821 | 0.98204 | 0.68 | 0.0393 | 1.08E-16 | 67.34018997 | 0.003656774 |
| MIP1b | 7 | rs1437220 | -0.1478 | -0.0020448 | 0.0497 | 0.055332 | 0.11 | 0.0315 | 3.53E-06 | 22.01545981 | 0.00206346 |
| MIP1b | 8 | rs1564708 | 0.1744 | 0.000621039 | 0.7296 | 0.758249 | 0.39 | 0.0188 | 2.87E-20 | 86.05522861 | 0.012000918 |
| MIP1b | 9 | rs17138331 | 0.1391 | 0.000318911 | 0.0944 | 0.119282 | 0.719999 | 0.0295 | 2.26E-06 | 22.23362252 | 0.003308207 |
| MIP1b | 10 | rs2411161 | -0.1714 | -0.00095011 | 0.0507 | 0.055022 | 0.450001 | 0.0367 | 3.14E-06 | 21.81169954 | 0.002827894 |
| MIP1b | 11 | rs281749 | -0.0799 | -0.0002324 | 0.7083 | 0.684439 | 0.7 | 0.0171 | 3.17E-06 | 21.83239287 | 0.002638015 |
| MIP1b | 12 | rs3760440 | -0.1236 | 0.000119955 | 0.331 | 0.340774 | 0.84 | 0.0162 | 2.75E-14 | 58.21124829 | 0.006765829 |
| MIP1b | 13 | rs62242409 | -0.1282 | -0.000542319 | 0.7425 | 0.754871 | 0.41 | 0.0193 | 3.43E-11 | 44.12263416 | 0.00628463 |
| MIP1b | 14 | rs72791296 | -0.2369 | -0.00044969 | 0.9414 | 0.953557 | 0.74 | 0.0466 | 3.78E-07 | 25.84391405 | 0.006192014 |
| MIP1b | 15 | rs72799710 | 0.1014 | 0.00112662 | 0.834 | 0.814825 | 0.12 | 0.0218 | 3.21E-06 | 21.63530006 | 0.002846951 |
| MIP1b | 16 | rs74810984 | -0.2206 | -0.000124825 | 0.0219 | 0.020379 | 0.95 | 0.0474 | 1.96E-06 | 21.65979455 | 0.002084819 |
| MIP1b | 17 | rs76582507 | -0.3175 | -0.000797275 | 0.9732 | 0.98215 | 0.719999 | 0.0677 | 3.26E-06 | 21.99429886 | 0.005258409 |
| MIP1b | 18 | rs76583883 | 0.2317 | 0.00418979 | 0.9682 | 0.958653 | 0.00340001 | 0.0511 | 4.99E-06 | 20.55939201 | 0.003305782 |
| MIP1b | 19 | rs76776296 | -0.2997 | -0.00101097 | 0.0368 | 0.038531 | 0.49 | 0.0598 | 5.55E-07 | 25.11719388 | 0.006367483 |
| MIP1b | 20 | rs9850846 | -0.072 | -0.000580444 | 0.4443 | 0.466854 | 0.31 | 0.0157 | 4.48E-06 | 21.03127916 | 0.002559833 |
| RANTES | 1 | rs112072646 | -0.4286 | 0.00246859 | 0.9692 | 0.972013 | 0.15 | 0.0862 | 6.48E-07 | 24.72235292 | 0.010967268 |
| RANTES | 2 | rs147509526 | 0.358 | 0.00123555 | 0.9871 | 0.984462 | 0.6 | 0.0717 | 6.93E-07 | 24.93031362 | 0.003263976 |
| RANTES | 3 | rs2251660 | -0.1829 | 0.000689835 | 0.1451 | 0.149502 | 0.39 | 0.0359 | 3.83E-07 | 25.95604472 | 0.008299275 |
| RANTES | 4 | rs4940620 | 0.2494 | 0.00044881 | 0.0636 | 0.061765 | 0.709999 | 0.054 | 3.54E-06 | 21.33071331 | 0.00740869 |
| RANTES | 5 | rs62438851 | 0.1957 | 0.000183462 | 0.1352 | 0.141 | 0.83 | 0.0414 | 2.33E-06 | 22.34503139 | 0.008955792 |
| RANTES | 6 | rs7000423 | 0.1318 | 0.00137211 | 0.674 | 0.322308 | 0.0239999 | 0.0253 | 1.82E-07 | 27.13874611 | 0.007633757 |
| RANTES | 7 | rs72793342 | 0.1487 | -0.00100636 | 0.7922 | 0.79915 | 0.16 | 0.0308 | 1.48E-06 | 23.30883159 | 0.007280016 |
| RANTES | 8 | rs74472919 | -0.3313 | -0.000501657 | 0.9811 | 0.966715 | 0.760001 | 0.0605 | 3.97E-08 | 29.98693805 | 0.004070502 |
| RANTES | 9 | rs75613039 | -0.37 | 0.000376015 | 0.9692 | 0.969087 | 0.82 | 0.081 | 4.81E-06 | 20.86572169 | 0.008173302 |
| RANTES | 10 | rs818452 | -0.2381 | -0.00129418 | 0.9484 | 0.925764 | 0.23 | 0.0505 | 2.36E-06 | 22.22982453 | 0.005548685 |
| TNFa | 1 | rs10834997 | 0.1247 | -0.000690501 | 0.3598 | 0.308785 | 0.26 | 0.0258 | 1.33E-06 | 23.36111111 | 0.007163739 |
| TNFa | 2 | rs111332265 | 0.3766 | -0.00185502 | 0.0716 | 0.058198 | 0.13 | 0.0754 | 6.63E-07 | 24.94697775 | 0.018855532 |
| TNFa | 3 | rs8121916 | -0.1306 | 0.000438361 | 0.7744 | 0.75361 | 0.5 | 0.0278 | 2.72E-06 | 22.06971689 | 0.00595965 |
| TNFb | 1 | rs10925040 | -0.1755 | -0.000520502 | 0.6113 | 0.621468 | 0.38 | 0.0373 | 2.67E-06 | 22.13790798 | 0.014637037 |
| TNFb | 2 | rs753274 | 0.1736 | -0.000273364 | 0.4642 | 0.45402 | 0.630001 | 0.0371 | 2.77E-06 | 21.89533642 | 0.014991231 |
| TNFb | 3 | rs7629875 | -0.3766 | -0.00063722 | 0.0577 | 0.051391 | 0.62 | 0.0774 | 1.37E-06 | 23.67438522 | 0.01542253 |
| TNFb | 4 | rs78296352 | -1.2215 | 0.000744327 | 0.9712 | 0.961221 | 0.61 | 0.1366 | 4.76E-21 | 79.96234906 | 0.083467633 |
| TRAIL | 1 | rs11618126 | -0.8908 | 0.000853978 | 0.0149 | 0.015607 | 0.709999 | 0.1914 | 1.46E-06 | 21.66090262 | 0.023294693 |
| TRAIL | 2 | rs11657269 | -0.1188 | -0.000537171 | 0.1193 | 0.156447 | 0.49 | 0.026 | 4.78E-06 | 20.87786982 | 0.002965728 |
| TRAIL | 3 | rs11699445 | -0.0746 | -0.000985995 | 0.3867 | 0.390552 | 0.089 | 0.0161 | 3.27E-06 | 21.46969639 | 0.002639701 |
| TRAIL | 4 | rs13115587 | -0.1019 | 0.000204495 | 0.9165 | 0.932091 | 0.86 | 0.0218 | 3.10E-06 | 21.84919199 | 0.001589269 |
| TRAIL | 5 | rs13185784 | -0.0846 | 0.000593728 | 0.7097 | 0.734459 | 0.36 | 0.0183 | 3.90E-06 | 21.37167428 | 0.002949121 |
| TRAIL | 6 | rs13278062 | -0.0801 | 0.000248476 | 0.497 | 0.493088 | 0.66 | 0.0157 | 3.57E-07 | 26.0294941 | 0.00320789 |
| TRAIL | 7 | rs146783010 | 0.6016 | 0.000178193 | 0.0109 | 0.01714 | 0.94 | 0.135 | 4.83E-06 | 19.85857668 | 0.007803912 |
| TRAIL | 8 | rs148051545 | 0.3921 | 0.00126864 | 0.9871 | 0.973015 | 0.49 | 0.0848 | 3.86E-06 | 21.37971837 | 0.003915386 |
| TRAIL | 9 | rs193112415 | 1.0421 | 0.00168746 | 0.0139 | 0.019085 | 0.42 | 0.0623 | 2.15E-62 | 279.7967712 | 0.029770392 |
| TRAIL | 10 | rs57396456 | 0.5626 | 2.98E-05 | 0.0338 | 0.031229 | 0.99 | 0.0518 | 1.25E-27 | 117.9614049 | 0.020673461 |
| TRAIL | 11 | rs62093514 | -1.0618 | -0.000744512 | 0.9742 | 0.972176 | 0.67 | 0.0552 | 6.86E-82 | 370.004739 | 0.056673922 |
| TRAIL | 12 | rs6878823 | 0.0879 | -0.000774417 | 0.7724 | 0.789072 | 0.27 | 0.0189 | 3.48E-06 | 21.62988158 | 0.002716579 |
| TRAIL | 13 | rs73039026 | 0.2999 | 0.00135716 | 0.0159 | 0.011842 | 0.6 | 0.0635 | 2.02E-06 | 22.30516709 | 0.002814617 |
| TRAIL | 14 | rs74488044 | -0.3489 | 0.00189001 | 0.9404 | 0.935338 | 0.1 | 0.0335 | 2.61E-25 | 108.4706705 | 0.013645543 |
| TRAIL | 15 | rs747324 | 0.0855 | 0.00108941 | 0.6412 | 0.680181 | 0.0729995 | 0.0178 | 1.61E-06 | 23.07237091 | 0.00336363 |
| TRAIL | 16 | rs74778900 | -0.5906 | -0.00132552 | 0.9861 | 0.98549 | 0.58 | 0.0532 | 2.59E-28 | 123.2433857 | 0.009562086 |
| TRAIL | 17 | rs75928541 | -0.275 | 0.000895908 | 0.9831 | 0.980174 | 0.66 | 0.0593 | 4.24E-06 | 21.50581972 | 0.002512926 |
| TRAIL | 18 | rs79287178 | 0.4317 | 0.000603983 | 0.9742 | 0.968859 | 0.719999 | 0.0421 | 9.12E-25 | 105.1477311 | 0.009368324 |

**Consent clearance copy**

**Declaration**

This document serves to clarify that the research project titled “Causal relationships between inflammatory cytokines and myopia: An analysis of genetic and observational studies” did not involve human participants in the method and data collection process, and the data used were all publicly available online, which are shared. Therefore, the requirement to obtain "consent to participate" from human subjects does not apply to this study. The nature of this study does not directly involve human subjects or include personal data from individuals.The ethics of animal experiments approval letter we behind in the page of the file is given.

We hereby declare that all activities undertaken in this research are in compliance with the ethical guidelines and adhere to the principles of integrity, respect, and responsibility towards scientific inquiry.

Furthermore, it is confirmed that this research project respects the dignity, rights, and welfare of any individuals or communities mentioned or implicated in the research findings indirectly. All data used is in the public domain, anonymized, or otherwise does not contain personally identifiable information.

**Consent and ethical clearance copy**


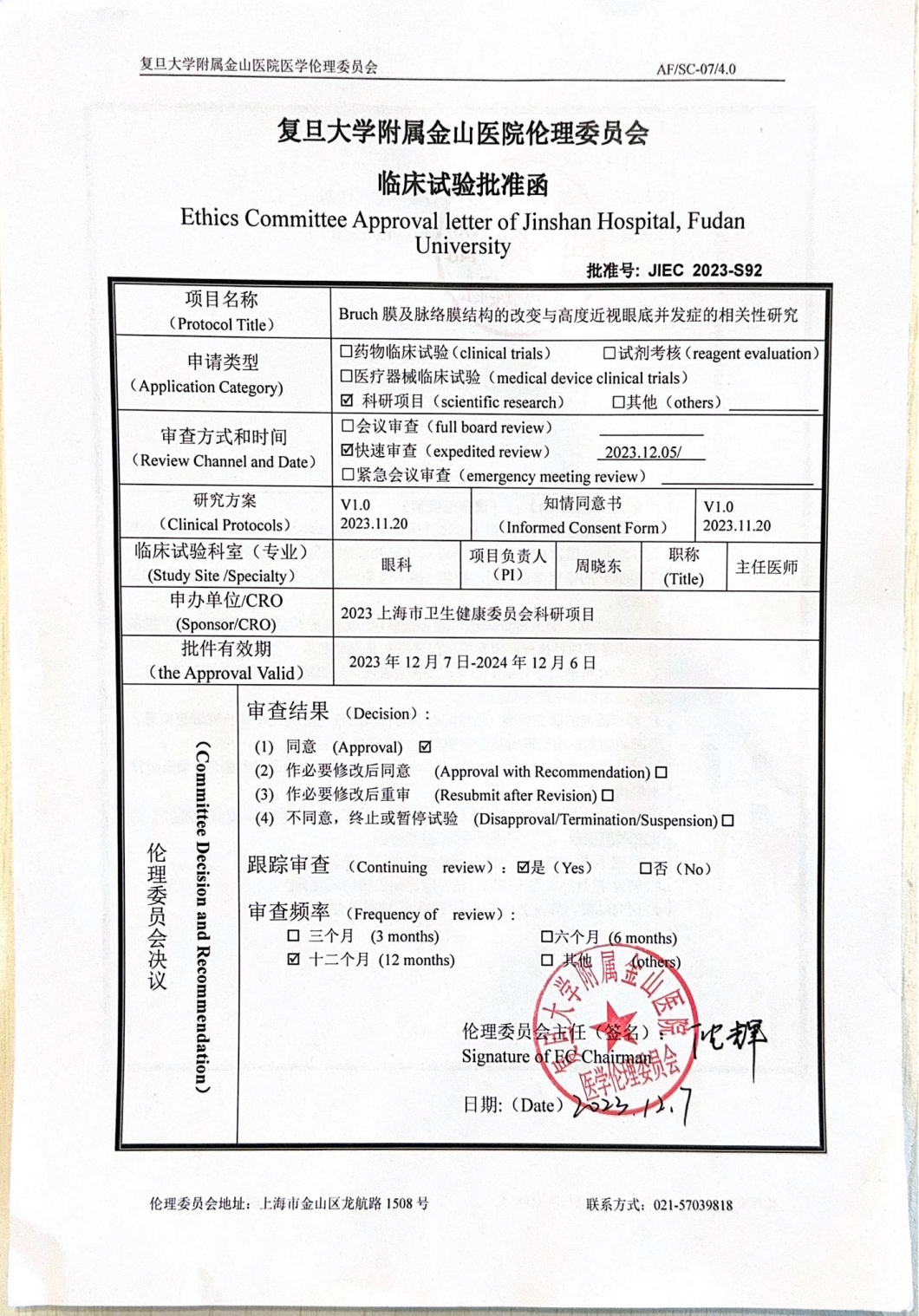


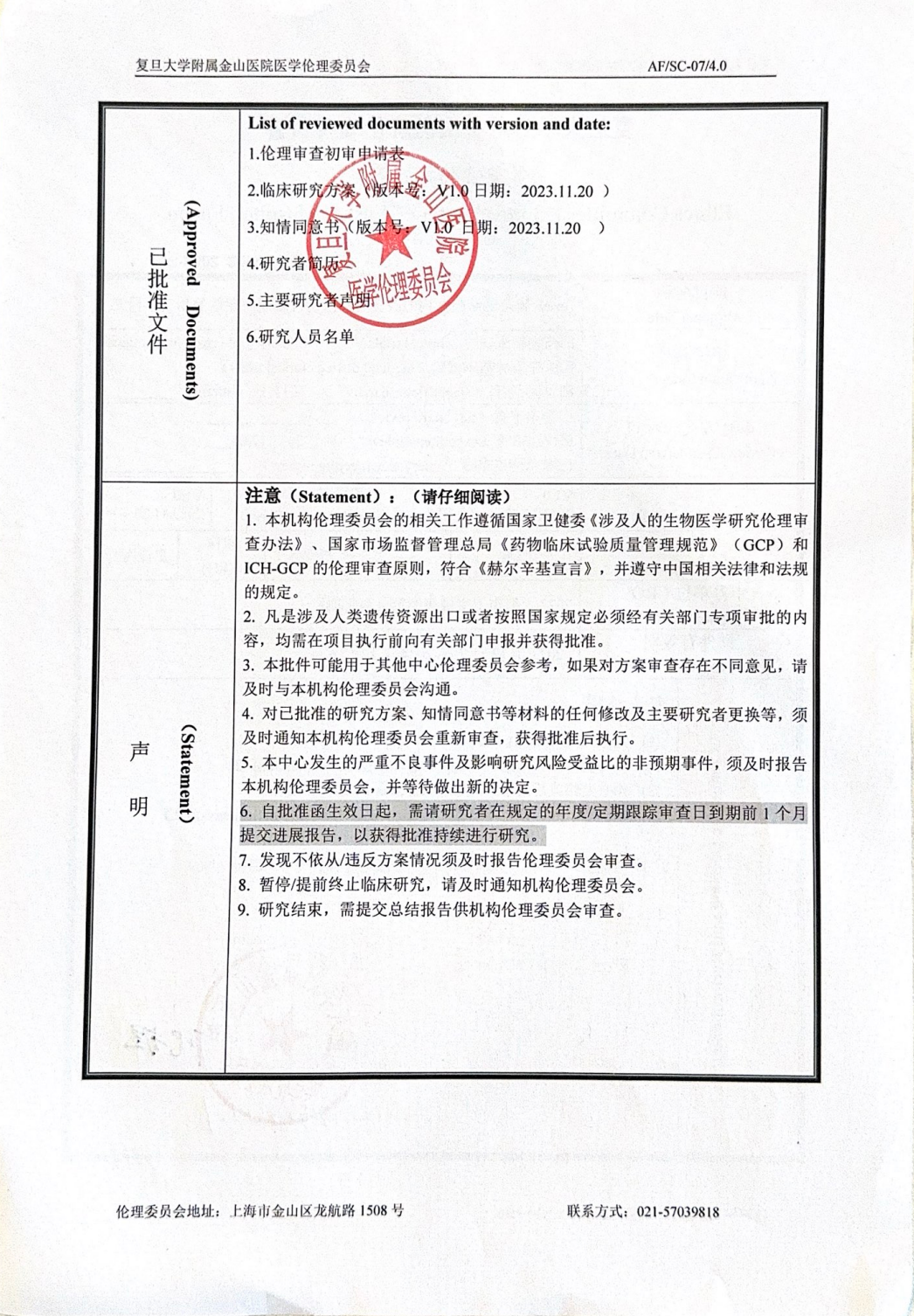

Supplement: Supplementary file 2 [file ms9-86-5179-s002.docx]
